# Physiological and molecular insight of microbial biostimulants for sustainable agriculture

**DOI:** 10.3389/fpls.2023.1041413

**Published:** 2023-01-30

**Authors:** Priya Kaushal, Nilofer Ali, Shivani Saini, Pratap Kumar Pati, Aparna Maitra Pati

**Affiliations:** ^1^ Biotechnology Division, CSIR-Institute of Himalayan Bioresource Technology, Palampur, HP, India; ^2^ Academy of Scientific and Innovative Research (AcSIR), Ghaziabad, India; ^3^ Department of Botany, Goswami Ganesh Dutta Sanatan Dharma College, Chandigarh, India; ^4^ Department of Biotechnology, Guru Nanak Dev University, Amritsar, Punjab, India

**Keywords:** PGPR, biostimulant, antioxidants, phytohormones, signaling, stress tolerance, crop productivity, systemic resistance

## Abstract

Increased food production to cater the need of growing population is one of the major global challenges. Currently, agro-productivity is under threat due to shrinking arable land, increased anthropogenic activities and changes in the climate leading to frequent flash floods, prolonged droughts and sudden fluctuation of temperature. Further, warm climatic conditions increase disease and pest incidences, ultimately reducing crop yield. Hence, collaborated global efforts are required to adopt environmentally safe and sustainable agro practices to boost crop growth and productivity. Biostimulants appear as a promising means to improve growth of plants even under stressful conditions. Among various categories of biostimulants, microbial biostimulants are composed of microorganisms such as plant growth-promoting rhizobacteria (PGPR) and/or microbes which stimulate nutrient uptake, produce secondary metabolites, siderophores, hormones and organic acids, participate in nitrogen fixation, imparts stress tolerance, enhance crop quality and yield when applied to the plants. Though numerous studies convincingly elucidate the positive effects of PGPR-based biostimulants on plants, yet information is meagre regarding the mechanism of action and the key signaling pathways (plant hormone modulations, expression of pathogenesis-related proteins, antioxidants, osmolytes etc.) triggered by these biostimulants in plants. Hence, the present review focuses on the molecular pathways activated by PGPR based biostimulants in plants facing abiotic and biotic challenges. The review also analyses the common mechanisms modulated by these biostimulants in plants to combat abiotic and biotic stresses. Further, the review highlights the traits that have been modified through transgenic approach leading to physiological responses akin to the application of PGPR in the target plants.

## Introduction

Agriculture is presently facing several challenges due to shortage of cultivable land, fluctuating weather conditions, increased incidence of pests and pathogens and rising weed infestations. To increase crop productivity, chemical fertilizers are used indiscriminately (Rouphael and Colla, 2020; Hendriksen, 2022). However, extreme usage of chemicals causes detrimental effects on the soil microorganisms, human and the environment leading to decreased water holding capacity, loss of soil fertility, imbalances in soil nutrients, and increased salinity levels (Wan et al., 2021; Jin et al., 2022). To meet the rising demand for food, boosting crop productivity is imperative. Hence there is a need for a green, efficient, sustainable and economically productive system to improve agronomic traits of crops (Drobek et al., 2019; Cataldo et al., 2022). Currently, biostimulants have emerged as one of the most potent and promising tools for enhancing the growth and productivity of crops naturally, simultaneously addressing the issues related to chemical fertilizers. Biostimulants/bioeffectors/bioprotectors or biobased products are different classes of organic or inorganic compounds which consists of bioactive substances or microorganisms and when applied on target plants promote its growth and productivity (Shahrajabian et al., 2021; Franzoni et al., 2022; Monteiro et al., 2022). Over the time, various researchers have classified biostimulants into nine broad categories including seaweeds and plant extracts, complex organic materials (obtained from sewage sludge extracts, composts, manure urban and agro-industrial waste products), humic substances, antitranspirants (kaolin and polyacrylamide), chitin and chitosan derivatives, elements (Al, Co, Se, Na, and Si), hydrolyzed proteins, nitrogen-containing compounds and microbial inoculants (Colla and Rouphael, 2020; Teklić et al., 2021; Franzoni et al., 2022; Monteiro et al., 2022). Out of this wide category, microbial-based biostimulants including plant growth-promoting bacteria (*Bacillus, Serratia, Arthrobacter, Pseudomonas, Rhodococcus, Enterobacter, Ochrobactrum, Acinetobacter, Azospirillum, Rhizobium, Streptomyces* and *Stenotrophomonas*) have surfaced as highly valuable and inexpensive agricultural input for improving plant yield (Orozco-Mosqueda et al., 2020; Baltazar et al., 2021; Miceli et al., 2021; Ayed et al., 2022; Fadiji et al., 2022). These microbiome based biostimulants trigger plant growth through solubilization of minerals (Zn, P and K), nitrogen fixation, production of phytohormones like indole-3-acetic acid (IAA), abscisic acid (ABA), ethylene (ET), cytokinin (CK), jasmonic acid (JA), secondary metabolites (siderophores, N-acyl homoserine lactone, lipopeptides, rhamnolipids, cyclic lipopeptides), enzymes (chitinases, cellulose, protease, glucanase etc.), volatile organic compounds (VOCs) (fatty acids and derivatives, hydrocarbons (alkanes, alkenes and alkynes), carbohydrates, (acids, alcohols, lactones, aldehydes, benzenoids, etc.) terpenoids, nitrogen (metalloid, amides, amines and imines), volatile inorganic compounds (HCN, H_2_S, NH_3,_ CO_2_, CO, and NO) and lipopolysaccharides (Caulier et al., 2019; Lephatsi et al., 2021). These components modulate root morphology including biomass of roots, its surface area and newly formed lateral roots, shoot length, leaf area, soil structure (nutrient, water holding capacity, porosity and water filtration), improve nutrient and mineral acquisition (N, P, Fe, Zn, Mn etc.) and photosynthetic capacity of a plant. Further, they also enhance biotic and abiotic stress tolerance by activating genes responsible for antioxidant defense system, production of phenolics, enzymes, amino acids and organic acids (Backer et al., 2018; Rouphael and Colla, 2018; Hamid et al., 2021; Aremu et al., 2022; Lin and Jones, 2022; Vocciante et al., 2022), but the exact functionality at cellular and biomolecular mechanisms are yet to be deciphered (Lephatsi et al., 2021; Othibeng et al., 2022). To elucidate the mechanism of action of biostimulants on plants, combined potential of molecular tools, proteomics, transcriptomics and metabolomics have been harnessed by several researchers (Franzoni et al., 2022). Although progress has been made in understanding the physiological and biochemical aspect of plant-microbe interactions under stress, but the core mechanism and elucidation of molecular interactions are still in infancy. Thus, the present review comprehends the physiological and biochemical modulations along with the signaling components of PGPR based biostimulants in imparting abiotic (major detrimental stresses like temperature, drought and salinity) and biotic stress tolerance in plants. An in-depth understanding of the biochemical and molecular mechanisms triggered by the microbial biostimulants particularly PGPR based will aid in designing and developing novel bioformulations for sustainable agriculture.

## Role of PGPR-based biostimulants in combating abiotic stress

### Drought and heat stress

**Figure 1:**
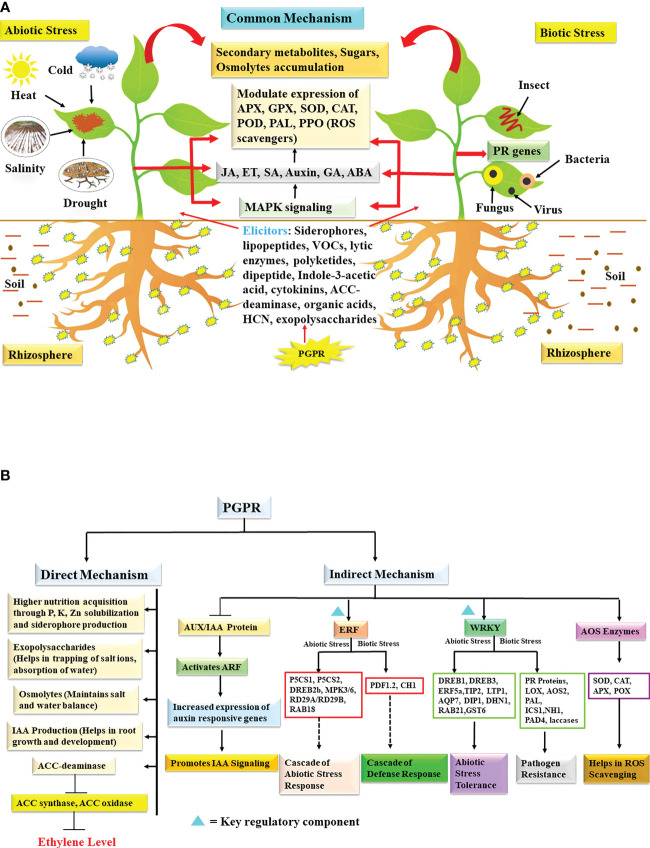
**(A)** Schematic representation of PGPR induced stress tolerance mechanism in plant challenged by abiotic and biotic stresses. Different elicitors released by PGPRs modulates endogenous phytohormones which in turn influences secondary metabolites, osmolytes production, activity of antioxidant enzymes and PR proteins. These combined metabolic pathways imparts stress tolerance and promotes plant growth under stressed environment. **(B)** PGPR-based direct and indirect mechanism involved in activating cascade of abiotic and biotic stress signaling in plants. The activation events are represented by arrows, inhibition process is represented by bar while dashed arrows represent signaling cascade. IAA, indole-3-acetic acid; ACC, 1-Amino Cyclopropane-1-Carboxylate; AUX/IAA, auxin/indole-3-acetic acid; ARF, auxin response factor; ERF, ethylene response factor; P5CS1, Δ^1^ - pyrroline-5-carboxylate synthase1; P5CS2, Δ^1^ - pyrroline-5-carboxylate synthase 2; DREB2b/DREB1/DREB3, drought-responsive element binding protein 2b, drought-responsive element binding protein 1, drought-responsive element binding protein 3; MPK3/MPK6, mitogen–activated protein kinase 3, mitogen-activated protein kinase 6; RD29A/RD29B, response-to-desiccation 29A, response-to-desiccation 29B; PDF1.2, protodermal factor 1.2; CH1, chitinase; WRKY, W-box domain binding transcription factor; TIP2, tonoplast intrinsic protein 2; LTP1, lipid transfer protein 1; AQP7, aquaporin 7; GST6, glutathione S-transferase; DIP1, dehydration stress-inducible protein 1; DHN1, dehydrin 1; RAB21, responsive to ABA protein 21; PR, pathogenesis related proteins; LOX, lipoxygenase; AOS2, allene oxide synthase 2; PAL, phenylalanine ammonia lyase; ICS1, isochorismate synthase 1; NH1, *Arabidopsis* NPR1 homolog 1; PAD4, p hytoalexin-deficient 4; AOS, antioxidant scavenging; SOD, superoxide dismutase; CAT, catalase; APX, ascorbate peroxidase; POX, peroxidases; ROS, reactive oxygen species.

About 60% of the world’s region falls under arid and semi-arid areas and depends mainly on irrigated agriculture (Swain et al., 2017). With climate change, it is expected that there will be a decrease in rainfall, a rise in temperature, an increase in atmospheric CO_2_ and severe alternations in weather conditions leading to frequent floods and droughts (Torres and Henry, 2018). In upcoming years, agriculture will be increasingly challenged by water scarcity, and plants will experience drought and heat stress leading to compromised productivity. Drought stress occurs under low humidity levels in soil, air and high ambient temperature (Lipiec et al., 2013; Kaya et al., 2020; Kosar et al., 2021; Mansoor et al., 2021), while heat stress can be described as an increase in temperature beyond a threshold measure hampering the normal development of a plant. It is observed that the combined action of both stresses restrict the physiological (photosynthesis, respiration, etc.), biochemical and cellular metabolism of plants such as cell membrane fluidity, integrity, elasticity, water potential, stomatal conductance, the structure of amino acids, proteins, nucleic acids, enzymes etc. (Rana et al., 2021; Mitra et al., 2021; Murali et al., 2021a; Noor et al., 2022; Shaffique et al., 2022). In order to adapt under environmental stress plants regulate their diverse molecular signaling pathways such as phytohormones, stress responsive proteins, antioxidants machinery, and osmolytes (Kosar et al., 2021). While understanding the physiological response of plants, it is crucial that both drought and heat stresses must be considered together as the physiological responses are closely interlinked and dependent (Dreesen et al., 2012). Numerous studies have elucidated the positive impact of PGPR functioning as a biostimulant on plants challenged by heat and drought (Rashid et al., 2022; Yasmin et al., 2022). Basically, drought and thermotolerance is a complex mechanism, however, microbial metabolites including organic acids, sugars, trehalose, choline, amino acids, proline, glycine betaine, polyamines, exopolysaccahrides (EPS), production of heat shock proteins (HSPs), dehydrins, VOCs, ACC-deaminase, phytohormones etc. play a vital role in imparting drought and heat tolerance [**Figures 1A, B**; (Ahluwalia et al., 2021; Ansari et al., 2021; Yasmin et al., 2021a; Notununu et al., 2022; Kour and Yadav, 2022; Shaffique et al., 2022)].

1-Amino Cyclopropane-1-Carboxylate (ACC) deaminase produced by several PGPRs help in combating drought stress experienced by its host plant by interfering with the ethylene biosynthesis pathway leading to lowering of ethylene concentration thereby counteracting stress signals. In one of the studies, the application of *B. licheniformis* K11 capable of producing auxin and ACC deaminase reduced the negative impact of drought in pepper without the use of agrochemicals (Lim and Kim, 2013). Similarly, improved growth was noticed in pea and maize under drought conditions on treating with ACC deaminase producing strain *Pseudomonas* spp., *Enterobacter cloacae*, *Achromobacter xylosoxidans* and *Leclercia adecarboxylata* primarily due to reduced ethylene accumulation as compared to untreated plants (Arshad et al., 2008; Danish et al., 2020). Additionally, these bacteria are capable of supplying nitrogen by sequestering and degrading ACC to α-ketobutyrate using ACC deaminase (Gupta and Pandey, 2019) thereby promoting better vegetative growth of plants.

Production of reactive oxygen species (ROS) is a common phenomenon observed under drought conditions causing damage to cells (Cruz de Carvalho, 2008) and antioxidant enzymes such as catalase (CAT), peroxidase (POD) and polyphenol oxidase (PPO) scavenge ROS preventing stress related injury thereby imparting stress tolerance [**Figures 1A, B**; (Zandalinas et al., 2018)]. These antioxidants also promote faster recovery from water limitation and dehydration compared to the control plants (Laxa et al., 2019). Hence, the potential of PGPR in enhancing the production of antioxidants is a desirable attribute. Several studies illustrate the beneficial trend of antioxidant enzymes under severe drought, as was noticed in mentha (Chiappero et al., 2019; Asghari et al., 2020) and in tomato (Mekureyaw et al., 2022). Noticeably, seeds of Pearl millet treated with *Bacillus amyloliquefaciens* (MMR04) enhanced expression of ascorbate peroxidase (APX) and superoxide dismutase (SOD) genes leading to the enhanced concentration of SOD and APX and decreased level of malondialdehyde (MDA) compared to the untreated lot (Murali et al., 2021b).

Accumulation of proline is one of the common mechanisms involved in imparting drought tolerance. Proline helps to maintain protein structure and activity thereby supporting membrane integrity (Kishor et al., 2005). Also, proline has multiple roles as it can act as a chelator of metals, a signaling molecule, and a defense molecule triggering the production of a series of antioxidants (Hayat et al., 2012). It has been observed that *Bacillus subtilis* (HAS31), *Pseudomonas* strains apart from increasing antioxidants (SOD, CAT and POD) activity also significantly enhanced the total soluble sugars and proline in potato and sweet corn (Batool et al., 2020; Zarei et al., 2020).

PGPRs have an inherent capability to produce plant growth hormones like auxin, gibberellins (GA), JA, salicylic acid (SA) and ABA. These hormones may be directly responsible for triggering a series of responses in the host (plant) to combat stress conditions. The role of SA in imparting drought stress has been illustrated to alter nitrogen metabolism, triggering the production of antioxidants and accumulation of glycine betaine thus conferring protection against stress (Khan et al., 2022 **)**. An interesting research showed that under heat-stress conditions inoculation of *Bacillus tequilensis* SSB07 strain in soybean enhanced the endogenous level of JA, SA, and reduced the level of ABA content (Kang et al., 2019a). All the treated plants had better biomass and photosynthetic pigment compared to the control. Two PGPR strains *Bacillus* sp. WM13-24 and *Pseudomonas* sp. M30-35, promoted the growth of ryegrass subjected to drought stress by modulating auxin distribution and ABA content in the plant (He et al., 2021).

Several scientific investigations illustrated that microbial applications induce the expression of specific genes related to drought response. Treating *Arabidopsis* and soybean with *Paenibacillus polymyxa* CR1 upregulated dehydration-responsive genes (*RD29A* and *RD29B*), which equipped the plants to face drought conditions (Liu et al., 2020). Similarly, application of *Bacillus subtilis* strain GOT9 triggered up-regulation of numerous drought stress related genes particularly *Response-to-desiccation 29B, 20* (*RD29B, RD20), RAB18* (encodes dehydrin protein)*, 9-cis- epoxycarotenoid dioxygenase (NCED3)*) in *Arabidopsis* and *BrDREB1D*, BrWRKY7 and *BraCSD3* in *Brassica* thereby minimizing the physiological damage. Enhanced expression of ABA inducible genes clearly showed that GOT9 increased ABA accumulation in plant and hence provided drought tolerance (Woo et al., 2020). Inoculation of *Pseudomonas putida* GAP-P45 in *Arabidopsis* modulated several important polyamine biosynthetic genes (*arginine decarboxylase (ADC)*, *agmatine iminohydrolase (AIH), N- carbamoyl putrescine amidohydrolase (CPA)*, *spermidine synthase (SPDS)*, *spermine synthase (SPMS)* and *S-adenosylmethionine decarboxylase (SAMDC)* thereby impacting cellular polyamine levels. The increased level of free cellular spermidine and putrescine positively correlated with water stress (Sen et al., 2018). In transgenic *Arabidopsis* overexpressing *ABA stress ripening 6 (OsASR6)* (auxin activated) gene increased the expression of auxin-responsive genes, *small auxin up-regulated family (SAUR32)*, Ser/Thr protein kinase (PINOID), and *auxin response factor 5 (ARF5)* auxin transcription factor leading to greater root density and biomass. These effects of *OsASR6* expression were found to mimic the beneficial effects of PGPRs in rice (Agarwal et al., 2019). PGPR strain *Streptomyces* mitigated drought stress in tomato and regulated the expression of transcription factors *ethylene response factor 1 (ERF1)* and *WRKY70* [**Figure 1B**; (Abbasi et al., 2020)]. In another study, expression of ABA independent genes, i.e. *drought-responsive element binding protein (DREB2* and *DREB1-2)* significantly enhanced due to PGPR (*Bacillus* sp.) inoculation in *Brassica* under water scarcity indicating *Bacillus*-mediated priming for drought tolerance (Bandeppa et al., 2019). Pepper treated with *B. licheniformis* K11 upregulated genes *Cadhn*, *VA*, *sHSP* and *CaPR10* leading to higher production of dehydrin-like protein, vacuolar H^+^-ATPase, small heat shock protein and pathogenesis-related protein which helped in in survival of plants under severe drought conditions (Lim and Kim, 2013). An interesting study was carried out wherein it was elucidated that *Pseudomonas putida* modulates the expression of important stress-responsive miRNAs in response to drought and salt stresses (Jatan et al., 2019).

All these studies illustrate that PGPRs influence expression of heat/drought related genes there by triggering production of series of antioxidants, osmolytes, proline and several key biomolecules which may contribute in mitigating heat and drought stress. Also, several hormones produced by PGPRs as illustrated in **Figure 1A** trigger cascade of biochemical reaction in host which enable plants to tie over drought stress.

### Cold stress

Low temperatures limit agriculture productivity in temperate ecosystems. In these areas, the plants constantly face chilling stress that leads to 51-82% annual yield loss (Hwarari et al., 2022). It generally affects the critical processes of plants such as ROS homeostasis, energy metabolism (electron transport chain), photosynthesis efficiency, cell wall structure, fluidity, root hydraulic conductance and structure of biomolecules (enzymes, proteins and nucleic acids) (Kazemi-Shahandashti and Maali-Amiri, 2018; Tiryaki et al., 2018; Zhou et al., 2021). In order to survive under prolonged low temperatures, the plants exhibit altered gene and transcription factor expression, leading to changes in membrane lipids, proteins, osmolytes levels, phytohormones, phenolic content and reactive oxygen scavenging enzymes (Brüggemann et al., 1999; Saltveit, 2000; Ait Barka et al., 2006; Amini et al., 2021; Guo et al., 2021; Ritonga et al., 2021; Saleem et al., 2021a; Eom et al., 2022; Hwarari et al., 2022; Wei et al., 2022).

The application of PGPR to improve crop productivity is a sustainable and safer means compared to chemical inputs. It has been found that treatment of *Burkholderia phytofirmans* strain *PsJN* (*Bp PsJN*) on *Arabidopsis thaliana* subjected to cold stress prevented disruption of plasmalemma, exhibited cell wall strengthening of mesophyll cells (Su et al., 2015). Inoculation of *Vitis vinifera* with PGPR strain *PsJN* exhibited better CO_2_ fixation, increased content of phenolics, proline and starch (Ait Barka et al., 2006). In another study, *PsJN* improved cold tolerance in *Vitis vinifera* wherein higher accumulation of proline, MDA and aldehydes (ALD) were observed along with higher expression of phenylalanine ammonia lyase (PAL) and stilbene synthase (STS) genes in primed plants compared to non-treated plants (Theocharis et al., 2012). Similarly, another finding revealed that under chilling stress treatment of tomato plants with psychrotolerant *Pseudomonas vancouverensis* (OB155) and *P. frederiksbergensis* (OS261) microbes minimized the stress effect by increasing the level of proline, antioxidant enzymes [(SOD, APX and glutathione (GSH)] (Subramanian et al., 2016). Further, *Pseudomonas fragi*, *P. chloropaphis*, *P. fluorescens* and *Brevibacterium frigoritolerans* inoculants also improved the growth of beans by regulating the activities of SOD, CAT, POX, APX and GR during low temperature stress [**Figure 1A**; (Tiryaki et al., 2018]. Subsequently, further research reflected the key role of *Bacillus* spp. in wheat under cold stress. Under cold stress, the bacterial treatment significantly reduced the level of ABA, ET, MDA by directly targeting the *ABA- response element* (*ABARE*), *Ethylene response factor* (*ERF*) and *4-Hydroxy-2-nonenal* (*4-HNE*; encodes α, β- unsaturated aldehyde during lipid peroxidation) genes, but at the same time the expression of *Δ^1^- pyrroline-5-carboxylate synthase* (*P5CS*), expansin (*expA1*), cytokinin (*CKX2*) and auxin (*ARF*) increased (Zubair et al., 2019). Another study evaluated the role of *Rhizobium* inoculation (RI) in legumes model plant i.e. *Medicago truncatula* against cold stress. Compared to the control plant, the treated plant showed a significant increase in the SOD, CAT, APX, ascorbate, reduced glutathione, proline, soluble sugars and glycine betaine, whereas POD, *lipoxygenase* (*LOX*) activity and nitro-oxidative damage was notably reduced. Moreover, RI also stimulated the nitrogen (N) uptake in cold stress seedlings by enhancing the activity of nitrate reductase (NR) enzyme (Irshad et al., 2021). In addition, *Streptomyces* sp. 506 (TOR3209) played a distinct function in boosting tolerance to cold stress in tomato. Expression profile of TOR3209 treated stressed plants showed an increased level of HY5 (bZIP) mediated ABA signaling genes [(*zeaxanthin epoxidase* (*ZEP1*), 9*-cis-epoxycarotenoid dioxygenase* (*NCED1*), carotenoid dioxygenase and carotene beta-hydroxylase), *dehydrin* (*TASI4*) (that triggers accumulation of soluble sugars, proline). Besides this, TOR3209 also reduced the photosynthetic damage by modulating the activities of RUBISCO (Ribulose 1, 5- bisphosphate carboxylase/oxygenase), NAD-MDH and NADP-MDH (malate dehydrogenases) enzymes suggesting that main mechanism of imparting cold tolerance is by ABA pathway (Ma et al., 2022). Over and above, other finding highlights the role of cold active PGPR in rice growth and development. It was observed that strains of *Pseudomonas*, *Enterobacter*, *Stenotrophomonas* genera inoculation ameliorated the effect of cold in rice by increasing the accumulation of metabolites (proline and soluble sugars), protein content, nutrients (N, P and K), antioxidants (SOD, POD and CAT) (Expósito et al., 2022).

These studies illustrate that most of the cold tolerance by PGPRs are due to cross-interlinkage of antioxidants, soluble sugars, proteins, proline, phenolics, phytohormones etc. Further scientific investigations will provide better insight into PGPR mediated cold tolerance in plants but nevertheless, there are credential evidences which indicate that the right microbial strains can boost productivity in the temperate ecosystem.

### Salinity stress

High salt concentration leads to osmotic stress that interferes with physiology, biochemical functioning (photosynthesis, stomatal conductance, enzyme activities, water and nutrient uptake), growth and yield of crops (Yasmin et al., 2021b; Choudhary et al., 2022). Microbial application triggers various mechanisms for improving plant growth under salinity stress (Sarkar et al., 2018). It generally includes the production of ACC-deaminase, EPS, phytohormones (auxin, CK and SA), antioxidant enzymes, VOCs, synthesis of osmoprotectant metabolites (proline, trehalose, alanine, glycine, glutamic acid, serine, threonine, aspartate, choline, betaine and organic acids), regulation of ion affinity transporters that in turn maintains ionic, osmotic, water homeostasis, thus resulting in improved plant growth under salt stress [**Figure 1A**; (Tewari and Arora, 2018; Abbas et al., 2019; Kumar et al., 2019; Bhat et al., 2020; Sunita et al., 2020; Choudhary et al., 2022; Gamalero and Glick, 2022; Kumawat et al., 2022)].

PGPR based compounds modulate the phytohormones, antioxidants and osmolytes levels in plants for their growth under stress conditions (Choudhary et al., 2022). A study demonstrated that under salt stress *Rhodopseudomonas palustris* G5 treated cucumber seedlings showed higher expression of SOD, POD, PPO and soluble sugars (Ge and Zhang, 2019) compared to untreated plants. Another ACC- deaminase producing endophytic strain *Pseudomonas* spp. OFT5 confers salt tolerance to tomato by reducing ethylene production (Win et al., 2018). Inoculating paddy with halotolerant *Curtobacterium albidum* strain SRV4 improved plant growth under various salinity levels by significantly increasing SOD, CAT, POX, APX expression, and by maintaining Na^+^/K^+^ homeostasis. This study showed that EPS production by strain reduced sodium ions availability to the plant and hence overcome effect of salinity stress (Vimal et al., 2019). Furthermore, *Planomicrobium* sp. MSSA-10 regulated antioxidants, phenolics and nutrients mobilization pathways in pea and promoted its growth in saline conditions. It was observed that bio-inoculant treatment increased total phenolics, POD, CAT and nutrients (N, P and K) uptake for reducing negative effects of salt [**Figure 1B**; (Shahid et al., 2018)]. Additionally, it was observed that salt tolerant *Bacillus pumilus* FAB10 significantly decreased antioxidant enzymes like SOD, CAT, glutathione reductase (GR) activities, proline and MDA content in wheat at different salt concentrations (Ansari et al., 2019). Furthermore, endophytic bacteria *Curtobacterium* sp. SAK1 treatment reduced the effect of salt stress in soybean by lowering endogenous ABA, JA, ROS, PPO and POD levels, whereas glutathione (cellular antioxidant) concentrations were found to be higher (Khan et al., 2019c). Another research team observed that inoculation of soybean with five halotolerant strains i.e. *Arthrobacter woluwensis* (AK1), *Microbacterium oxydans* (AK2), *Arthrobacter aurescens* (AK3), *Bacillus megaterium* (AK4) and *Bacillus aryabhattai* (AK5) conferred salt tolerance by elevating the expression of SOD, glutathione synthase (GSH) and enhancing K^+^ uptake. Besides the antioxidants, microbial inoculation significantly reduced Na^+^ ion concentration, ABA level, but increased the expression of IAA related gene i.e. *auxin resistant 1 *(*GmLAX3*) and salt tolerant gene (*Soybean salt tolerance 1*) (*GmST1*) (Khan et al., 2019b). In addition to this, it was found that halotolerant bacteria *Leclercia adecarboxylata* MO1 improved tomato growth under salt stress by significantly increasing sugars (sucrose, glucose and fructose), organic acids (citric acid and malic acid), amino acids (serine, glycine, methionine and proline) and simultaneously decreasing endogenous ABA level (Kang et al., 2019b). Similarly, PGPR *Pseudomonas* PS01 imparted salt tolerance in *Arabidopsis* by modulating the expression of stress related genes. Results illustrate that PS01 inoculation in salt stressed plants increased expression of *lipoxygenase (LOX2)* (related to JA synthesis), while decreased *APX2*, *GLY17* (ROS scavenging and detoxification). No significant change was observed in the expression of *RD29A* and *RD29B* (ABA signaling genes) (Chu et al., 2019). One more study figured out that in rice inoculation with halotolerant *Glutamicibacter* sp. YD01 increased the expression of antioxidants such as *OsPOX1*, *OsFeSOD*, *OsGR2*, abiotic stress related genes (*OsWRKY1*, *OsDREB2*), *OsHKT1* (related to ionic balance) and downregulated *OsERF1* (related to ethylene production), thus enhancing their tolerance to salt stress (Ji et al., 2020). In a different fascinating research it was found that *Pseudomonas pseudoalcaligenes* (SR16) and *Bacillus subtilis* (SR3) also provide salt stress tolerance to hydroponically grown soybean seedlings. The strain SR16 effectively reduced 100mM NaCl stress by increasing the total protein, proline content, and activities of various antioxidants (SOD, CAT, APX, POD, PAL and PPO) (Yasmin et al., 2020). Recent findings suggest that soil application of *Kosakonia sacchari* improved mung bean performance by reducing the level of oxidative stress markers such as proline, MDA, H_2_O_2_ content, antioxidants like APX, CAT, SOD and GR. In contrast, the level of antioxidant metabolites i.e. ascorbic acid and glutathione increases in the foliage of treated plants which in turn reduced the toxicity of NaCl (Shahid et al., 2021). Moreover, inoculation of salt-tolerant PGPR *Acinetobacter johnsonii* provided tolerance to maize by downregulating SOD, CAT, proline and MDA content. It was observed that rhizobacterial inoculation improved dehydrogenase, alkaline phosphatase, acid phosphatase, urease and enzyme activity in the soil. Improved microbes mediated soil enzyme activities plays an important role in balancing nutrient profile, plant growth under salt stress (Shabaan et al., 2022). Additional evidence demonstrates role of halotolerant *Bacillus* strains (NMCN-1 and LLCG23) in mitigating 200mM salinity stress in wheat. Application of both inoculants significantly downregulated the expression of *ABA- response element* (*ABARE*), *4-Hydroxy-2-nonenal* (*4-HNE*), whereas the *P5CS* gene was found to be upregulated. This clearly illustrates that these halophilic microbes regulate key stress signaling pathways (ABA synthesis, MDA and proline production) that subsequently lowered the effect of stress (Ayaz et al., 2022). From the cited literature (**Table 1**), it is evident that PGPR could alleviate salt stress by improving soil health, nutrient uptake, hormone production, antioxidant activity, and stress- responsive genes.

**Table 1:** PGPRs along with their mode of action in combating abiotic stress.

PGPR Strain	Host Plant	Stress	Mechanism of action	References
*Pseudomonas aeruginosa*	Sorghum	Heat	Increased proline, chlorophyll, sugar, amino acids, and protein content	Ali et al. (2009)
*Rhizobium, Pseudomonas*	Maize	Salinity	Decreased electrolyte leakage and increased proline, relative water content	Bano and Fatima (2009)
*Pseudomonas putida*	Wheat	Heat	Reduced membrane injury and the level of antioxidant enzymes such as SOD, APX and CAT	Ali et al. (2011)
*Pseudomonas fluorescens*, *Bacillus subtilis*	Green Gram	Water	Enhanced activity of *CAT1* and POD	Saravanakumar et al. (2011)
*Pseudomonas chlororaphis*	*Arabidopsis thaliana*	Drought	Up-regulation of genes such as *NIT1* (associated with plant growth regulators), *Atcor15a* (associated with ABA), *RD21a*, KIC (calcium binding protein**)**	Cho et al. (2011)
*Pseudomonas koreensis, Pseudomonas fluorescens, Pseudomonas jessenii*	Wheat	Cold	Increased relative water content, anthocyanin, proline, total phenolics, starch and reduced electrolyte leakage	Mishra et al. (2011)
*Bacillus licheniformis*	Pepper	Drought	Increased content of stress proteins i.e. *Cadhn*, *VA*, *sHSP* and *CaPR*-10	Lim and Kim (2013)
*Bacillus pumilus, Bacillus firmus*	Potato	Salinity/Drought	Enhanced mRNA expression related to ROS scavenging enzymes (SOD, GR, CAT, DHAR and APX) and proline level	Gururani et al. (2013)
*Bacillus megaterium, Bacillus subtilis*	Wheat/Barley	Cold	Significant reduction in the level of ROS and antioxidant enzyme (SOD, POD and CAT)	Turan et al. (2013)
*Pseudomonas aeruginosa*	Mung bean	Drought	Increased expression of CAT, POX, SOD and drought responsive genes (*DREB2A*, *CAT1* and *DHN*)	Sarma and Saikia (2014)
*Bacillus amyloliquefaciens, Azospirillum brasilense*	Wheat	Heat	Increased level of DHAR (Dehydroascorbate reductase), MDHAR (Mono-dehydroascorbate reductase) and GR whereas decreased APX and modulated expression of *SAMS1*, *HSP17*.8	Abd El-Daim et al. (2014)
*Enterobacter* sp.	Arabidopsis	Salinity	Up-regulated expression of salt stress responsive genes such as *DREB2b*, *RD29A*, *RD29B*, and RAB18, proline biosynthesis genes (*P5CS1* and *P5CS2*), MPK signaling genes (*MPK3* and *MPK6*)	Kim et al. (2014)
	Tomato		Enhanced expression of APX	
*Proteus penneri, Pseudomonas aeruginosa, Alcaligenes faecalis*	Maize	Drought	Increased relative water content, sugar, decreased proline, antioxidant enzymes (SOD, POD and CAT)	Naseem and Bano (2014)
*Burkholderia phytofirmans*	*Arabidopsis thaliana*	Salinity	Enhancement of the proline and transcript level of ABA signaling genes (*RD29*, *RD29B), APX2 (*antioxidant related), *GYLI*7 (glyoxylate pathway), decreased expression of *LOX2* (related to JA)	Pinedo et al. (2015)
*Bacillus megaterium, Enterobacter* sp.	Okra	Salinity	Increased ROS scavenging enzymes (CAT, SOD, APX, GR and DHAR)	Habib et al. (2016)
*Bacillus pumilus*	Rice	Salinity	Augmented activity of antioxidants such as (SOD, POD and CAT)	Khan et al. (2016)
*Dietzia natronolimnaea*	Wheat	Salinity	Enhanced expression of salt stress tolerant (*TaST)*, Salt Overly Sensitive (SOS) related genes (*SOS1* and *SOS4*), antioxidant enzymes genes (*APX, MnSOD, CAT, POD, GPX* and *GR*) and ABA signaling **(** *TaABARE* and *TaOPR1)*	Bharti et al. (2016)
*Pseudomonas frederiksbergensis, Flavobacterium glaciei, Pseudomonas vancouverensis*	Tomato	Cold	Increased proline content and, enhanced antioxidant enzymes such as SOD, APX and GSH	Subramanian et al. (2016)
*Bacillus aryabhattai*	Soybean	Heat	Enhanced levels of phytohormones IAA, JA, GA, ABA, antioxidants (CAT and SOD), but CK decreased	Park et al. (2017)
*Bacillus megaterium*	Arabidopsis	Salinity	Enhanced level of *CYP94B3* (responsible of JA-Ile catabolism), MDHAR and ATP synthase	Erice et al. (2017)
*Bacillus subtilis*	Wheat	Salinity	Decreased proline, MDA content whereas SA and water storage capacity enhanced	Lastochkina et al. (2017)
*Bacillus amyloliquefaciens*	*Arabidopsis thaliana*	Salinity	Up-regulation of genes such as *GST* and *POX* **(**antioxidant responsive), *ACS7*, *ACS2*, *ACS8*, and *ACS*11(ET signaling), *LOX* (JA signaling) downregulated *NCED3, NCED4, ABI1* and *MARD1* (ABA signaling)	Liu et al. (2017)
*Pseudomonas chlororaphis, Pseudomonas extremorientalis*	Tomato	Salinity	Decreased H_2_O_2_, APX, GR level with simultaneous increase in SOD and CAT activity	Egamberdieva et al. (2017)
*Azospirillum brasilense, Herbaspirillum seropedicae*	Maize	Drought	Decreased expression of *ZmVP14* (involved in the biosynthesis of ABA), proline, ET content but MDA level increased	Curá et al. (2017)
*Pseudomonas putida*	Finger Millet	Drought	Increased activities of SOD, CAT, APX and GPX antioxidants	Chandra et al. (2018)
*Paraburkholderia phytofirmans*	Tomato	Heat	Augmented chlorophyll content, gas exchange, expression of *APX2* and *CAT1.* No significant change was observed in SOD, *CHI3*, *TIV1*, *Frk2, Hxk1*, *Hxk2*, *RbcL* and *RbcS* level	Issa et al. (2018)
*Ochrobactrum pseudogrignonense, Pseudomonas* sp., *Bacillus subtilis*	Black gram/Pea	Drought	Downregulated expression of *ACO*, increased proline content and, antioxidant enzymes (CAT and POD)	Saikia et al. (2018)
*Pseudomonas fragi, Pseudomonas chloropaphis, Pseudomonas fluorescens, Brevibacterium frigoritolerans*	Bean	Cold	Decreased MDA, ROS (O_2_ and H_2_O_2_) and POX level whereas SOD, CAT, APX and GR activity increased	Tiryaki et al. (2018)
*Arthrobacter woluwensis*	Soybean	Salinity	Upregulated expression of salt stress response genes such as *GmLAXs* and *GmST*, Low level of ABA and JA. Significant change in the activities of PPO and POD was also observed	Khan et al. (2019a)
*Ochrobactrum pseudogrignonense*	Wheat	Salinity	Increased activity of APOX, GR, SOD and germin-like proteins, whereas no significant change in the level of POX and CAT	Chakraborty et al. (2019)
*Bacillus pumilus*	Wheat	Salinity	Reduced antioxidant enzyme (CAT, SOD, GR) activities, proline and MDA content	Ansari et al. (2019)
*Bacillus velezensis*	Wheat	Heat/Cold/ Drought	Modulated various stress related proteins, antioxidant activity, amino acids metabolic pathways and accumulation of γ-aminobutyric acid (GABA)	Abd El-Daim et al. (2019)
*Pseudomonas fluorescens, Bacillus amyloliquefaciens*	Peppermint	Drought	Increased total phenolic content, antioxidant enzymes (SOD and POX), reduced proline and MDA content	Chiappero et al. (2019)
*Bacillus* sp.	Guinea grass	Drought	Reduced proline accumulation GR activity and increased APX level	Moreno-Galván et al. (2020)
*Cupriavidus necator, Pseudomonas fluorescens*	Maize	Drought	Increased nitrogen and phosphorous use efficiency	Pereira et al. (2020)
*Azotobacter chroococcum, Azospirillum brasilense*	Peppermint	Drought	Augmented ABA, SOD, proteins, soluble sugars, phenolic, flavonoid and oxygenated monoterpenes, but other antioxidant enzymes GPX and CAT activity decreased	Asghari et al. (2020)
*Kocuria rhizophila*	Maize	Salinity	Increased antioxidant enzyme (APX, GPX and GR), proline and expression of *ZmGR1, ZmAPX* (encoding antioxidants), *ZmNHX1*, *ZmNHX2, ZmNHX3, ZmWRKY58* and *ZmDREB2A (*salt tolerance genes*)*, whereas decreased MDA	Li et al. (2020)
*Bacillus cereus*	Soybean	Heat	Increased APX, SOD, GSH, proline, expression of *GmLAX3*, *GmAKT2* (genes involved in the regulation of the ABA). Decreased MDA content and expression of *GmHSP* (heat shock protein)	Khan et al. (2020)
*Bacillus cereus*	Tomato	Heat	Increased proline content, and antioxidant enzymes (SOD, POD and CAT)	Mukhtar et al. (2020)
*Bacillus cereus, Serratia marcescens, Pseudomonas aeruginosa*	Wheat	Salinity	Decreased antioxidant enzymes (SOD, CAT and POX), non-enzymatic antioxidants (GSH, AsA, and α-TOC)	Desoky et al. (2020)
*Bacillus* sp.	Rye grass	Drought	Increased proline, antioxidant enzymes (CAT and POD), decreased MDA, relative membrane permeability and H_2_O_2_ accumulation	He et al. (2021)
*Bacillus sonorensis, Bacillus cereus, Bacillus subtilis, Bacillus safensis, Bacillus paramycoides, Bacillus tequilensis, Brevibacillus* sp.	Cotton	Salinity	Increased absorption of K^+^, while decreased absorption of Na^+^ and, maintenance of the proline content, Chlorophyll Content Index (CCI), Relative Water Content (RWC) and Relative Electrolyte Leakage (EL)	Saleem et al. (2021b)
*Pseudomonas putida*, *Alcaligenes* sp., *Klebsiella* sp., *Pseudomonas cedrina*	Alfalfa	Salinity	Reduced proline, MDA and H_2_O_2_ level	Tirry et al. (2021)
*Bacillus megaterium, Bacillus licheniformis*	Wheat	Drought	Increased proline, and antioxidant enzymes (SOD, CAT, APX, POD and GR)	Rashid et al. (2022)
*Bacillus subtilis, Bacillus pumilus*	Cotton	Salinity	Modulated the ascorbate, aldarate, glyoxylate, dicarboxylate metabolism pathways, and pentose, glucuronate interconversions pathway	Akbar et al. (2022)
*Bacillus subtilis, Pseudomonas* sp.	Brinjal	Salinity	Increased level of free polyamines (spermine, spermidine, puterscine), expression of *psbD*, GR, GST and Protease I/II whereas lipases level decreased	Mokabel et al. (2022)
*Bacillus thuringiensis* PM25	Maize	Salinity	Increased antioxidants (APX, POD, SOD, AsA), total soluble sugars, proteins, flavonoids, osmolytes (free amino acids, glycine betaine and proline)	Ali et al. (2022)
*Bacillus butanolivorans*	Pepper	Drought	Increased expression of *P5CS*, *P5CR (*proline synthesis genes), *Cadhn*, *sHSP* (drought-sensitive gene), *bZIP1 (*ABA-related genes), *LOX, COI1* (JA-related genes), POX, glutathione, but decreased CAT and SOD	Kim et al. (2022)

From the above studies it is clear that bacterial production of ACC deaminase (lowers ethylene concentration), enhanced production of a range of antioxidants (scavenges ROS) and higher production of proline (signaling molecule) in stress affected plants are some of the common mechanisms operate in PGPR treated plants to enable them combat temperature, drought and salinity stress. Hence, application of PGPR based biostimulants with proven PGP traits could be an ecofriendly sustainable means to boost crop productivity under single or combined abiotic stress.

## Role of PGPR-based biostimulants in imparting tolerance against disease (biotic stress)

Plant resistance against pathogens is generally based upon two mechanisms i.e. induced systemic resistance (ISR) and systemic acquired resistance (SAR). ISR is mainly mediated by beneficial microorganisms through root colonization, root immunity modulation and production of certain elicitors like siderophores, polysaccharides, VOCs, plant hormones, enzymes etc. whereas SAR is defined as the plant’s acquired or adaptive resistance (Olowe et al., 2020; Hamid et al., 2021). Both ISR and SAR resistance mechanism is effective against wide group of pests and pathogens (Vlot et al., 2021; Meena et al., 2022; Yu et al., 2022). Though numerous studies reported that PGPR modulates various physiological, biochemical and molecular processes in plants and helps their survival under pathogens attack (Olowe et al., 2020; Castiglione et al., 2021; Yu et al., 2022), but the core mechanism of action is not fully understood. The results obtained through systematic studies indicate that induction of resistance against multiple pathogens including virus, fungi, bacteria rely on combined mechanisms that may work simultaneously (Yu et al., 2022). It includes induction of specific defense response genes/enzymes like ROS scavengers/antioxidants such as (CAT, APX, guaiacol peroxidase (GPX), GR, POD and SOD), accumulation of phytohormones (JA, ET, SA, GA and auxin), glucanases, chitinases, sugars, osmolytes, pathogenesis related proteins (PR) and secondary metabolites which in turn are directly involved in controlling growth and proliferation of pathogen [**Figures 1A, B**; (Baxter et al., 2014; Pieterse et al., 2014; Conrath et al., 2015; Camejo et al., 2016; Li et al., 2016; Guo et al., 2019; Ebrahimi et al., 2020; Olowe et al., 2020; da Silva et al., 2021; Luo et al., 2022)].

From the wide group of PGPR, *Bacillus* sp. has been considered as an excellent agent for controlling pathogens attack in various plants such as tomato, banana, tobacco, rice, wheat, cucumber, watermelon, cotton (Saechow et al., 2018; Gamez et al., 2019; Wu et al., 2019; Fu et al., 2020; Jiao et al., 2020; Dimopoulou et al., 2021; Kazerooni et al., 2021; Luo et al., 2022). *Bacillus amyloliquefaciens* (SN13) is a bio-protective agent against *Rhizoctonia solani* (causative agent of sheath blight disease), which enhances defense response in the rice plants. The colonized plants showed alteration in phytohormone content (increased level of SA, ABA and GA), and MAPKinases (increased level of phospholipase D and serine-threonine protein kinases) signaling pathways that helped in controlling disease proliferation. Apart from these, SN13 treatment also modulated the production of secondary metabolites (quinazoline) and ROS regulators (arabitol, proline and mannitol, sugars like β-D-glucopyranose, fructopyranose, and myoinositol, ferric reducatse glutathione *S*-transferase and peroxidase precursor) (Srivastava et al., 2016). Moreover, cotton (*Gossypium hirsutum*) plants treated with blend of *Bacillus* spp. enhanced secretion of gossypol (allelochemical) and JA (defense related phytohormone) which in turn reduced larval feeding of *Spodoptera exigua* (beet armyworm). In addition, treated cotton plants exhibited increased expression of genes involved in synthesis of allelochemicals i.e. *(+)-δ-cadinene synthase* (*CAD1* gene family, including *Cad1- C1, Cad1-A, Cad1-C14 and Cdn-C3*) and jasmonates *(allene oxide synthase*) (*GhAOS), 13-lipoxygenase (GhLOX1)* and *12-oxo-phytodienoicacid reductase 3* (*GhOPR3* [**Figure 1A**; (Zebelo et al., 2016)]. Another rhizobacterium strain *Bacillus amyloliquefaciens* SQRT3 strongly inhibited tomato bacterial wilt disease (caused by *Ralstonia solanacearum*). The application of SQRT3 increased the expression of POD, PPO, stress marker genes like *proteinase inhibitor 2* (*PIN2*) (related to JA pathway) and *pathogenesis related protein-1a (PR-1a*) (related to SA pathway) and *Omsotin-like* (related to ET pathway) (Chunyu et al., 2017). Likewise, *Bacillus velezensis* enhanced resistance of pepper plants against *Botrytis cinerea* BC1301 (causative agent of gray mold disease) by triggering antioxidants SOD, CAT, POD and SA- mediated defense signaling genes namely *non-expressor of pathogenesis-related genes 1* (*NPR1*)*, pathogenesis related protein-1 (PR1)* and *peroxidase*. However, no effect was observed in the expression of *proteinase inhibitor 2 (PIN2)* and (*TIN1)* genes (Jiang et al., 2018). In another study, anti-pathogenic role of *Bacillus amyloliquefaciens* Ba13 against tomato yellow leaf curl virus (TYLCV) was observed. It was found that inoculation of this beneficial microbe improved tomato growth by elevating the expression of systemic resistance related genes including *pathogenesis related protein-1, 2, 3 (PR1*, *PR2* and *PR3*), chitinase, PAL, POD, PPO, and β-1,3-glucanase (Guo et al., 2019). Furthermore, an interesting work underline the effectiveness of *Bacillus amyloliquefaciens* YN201732 against tobacco powdery mildew disease (caused by *Erysiphe cichoracearum*). Results highlight that bacterial treatment inhibited pathogenic fungi growth over tobacco cultivar by increasing the expression of disease-related genes like *non-expressor of pathogenesis-related genes 1 (NPR1), plant defensin 1.2 (PDF1.2), chitinase (chit)* and PPO, whereas, no significant change was observed in POD and PAL activity (Jiao et al., 2020). Also, *Enterobacter asburiae* BQ9 imparted tolerance against tomato yellow leaf curl virus by enhancing the expression of antioxidant enzymes such as POD, CAT, PAL and SOD; defense-related genes i.e. *pathogenesis related protein-1a, 1b* (*PR1a* and *PR1b*) (Li et al., 2016). These findings illustrate that treatment with PGPR may activate biochemical and molecular changes to restrict pathogenic invasions in plants. Additionally, in *Nicotiana tabacum* cv. plants *Peanibacillus lentimorbus* B-30488 inoculation reduced cucumber mosaic virus (CMV) RNA accumulation by ~12 fold (91%). This ISR was linked with an increase in expression of stress related genes *Brassinosteroid signaling kinase 1* (*BR-SK1*)*, RNA dependent RNA polymerase 2* (*RdRP2*)*, zinc finger – homeodomain (ZF-HD), pathogenesis related protein 1(PR1), β-1,3-glucanase* (*Gluc), asparagine synthetase (AsSyn), tetrahydrocannabinolic acid synthase* (*TCAS*) and antioxidant enzyme (APX, GPX, SOD and CAT) (Kumar et al., 2016). Similarly, *Peanibacillus lentimorbus* B-30488 also provides resistance against southern blight (caused by *Scelerotium rolfsii)* disease in tomato. The treated plants showed alteration of the ET pathway by significantly suppressing *1-aminocyclopropane-1-carboxylate synthase (ACC synthase)* and *oxidase (ACO)* enzymes, and antioxidant enzyme activities (APX, GPX and SOD); whereas systemic tolerance was associated with expression of *pathogenesis –related protein -1, 2A, 4, 7* (*PR1, PR2A, PR4* and *PR7)*, CAT, *chitinase (CHI3* and *CHI9), β-1, 3- glucanase* (*GLU*), calmodulin and PPO (Dixit et al., 2016). Another report elucidates the role of *Pseudomonas aeruginosa* in controlling fungus (*Botrytis cinerea*) infection in *Brassica napus* by inducing the expression of transcription factor (*BnWRKY33),mitogen-activated protein kinase 3, 4 (BnMPK3* and *BnMPK4*) and *pathogenesis related protein-1 and 4 (BnPR1* and *BnPR4*) (Monnier et al., 2018).

Apart from this, some PGPRs also impart resistance against pathogens by secreting anti-microbial compounds (Hamid et al., 2021; Ji et al., 2021). For example, different *Bacillus* strains such as *Bacillus velezensis* QST713*, Bacillus velezensis* (C2), *Bacillus velezensis* OEE1, *Bacillus subtilis* release antifungal compounds such as lipopeptides (fengycin, bacillomycin and surfactin), polyketides (bacillaene, macrolactin and difficidin), dipeptide bacilysin, antifungal VOCs (phenylethyl alcohol, benzeneacetic acid, benzaldehyde, 1-decene, tetradecane) and lytic enzymes (chitinase, protease and β-glucanase), 3-indolylacetonitrile and suppresses green mold (caused by *Trichoderma aggressivum* f*. europaeum*), Verticillium wilt disease (caused by *Verticillium dahliae*), Septoria tritici blotch (caused by *Zymoseptoria tritici*), in button mushroom, tomato, olive, wheat (Mejri et al., 2018; Pandin et al., 2018; Dhouib et al., 2019; Azabou et al., 2020). Moreover, a research illustrated that application of *Herbaspirillum seropedicae* (BAC) suppressed *Xanthomonas euvesicatoria* (Xe), a causative agent of bacterial spot disease in ‘Micro-Tom’ *Solanum lycopersicum* L by downscaling the concentration of various organic acids such as oxalic acid, succinic acid, citric acid that enhances pathogens virulence (da Silva et al., 2021). The compiled studies (**Table 2**) clearly illustrate that PGPRs help in imparting tolerance against pathogen by activating pathway(s) that leads to production of array of defense related metabolites in plants.

**Table 2:** PGPRs along with their mode of action in combating biotic stress.

PGPR Strain	Host Plant	Pathogen	Disease	Mechanism of action	Benefits	References
*Bacillus amyloliquefaciens* (SN13)	Rice	*Rhizoctonia solani*	Sheath blight	Target phytohormones (SA, ABA, GA), MAPK, ROS signaling. Increased secondary metabolite production	Reduces fungal dry mass, number, size, length and diameter of spot lesions	Srivastava et al. (2016)
*Bacillus* sp.	Cotton	*Spodoptera exigua*	–	Increased gossypol and JA production	Significant reduction in larvae mortality rate	Zebelo et al. (2016)
*Enterobacter asburiae* BQ9	Tomato	*Tomato yellow leaf curl virus*	–	Enhanced expression of *PR1a, PR1b*, POD, CAT, POL and SOD	Milder disease symptoms (stunting, yellowing, curling of leaves)	Li et al. (2016)
*Peanibacillus lentimorbus* B-30488	Tobacco cv.	*Cucumber mosaic virus*	–	Targets PR genes and antioxidant enzymes	Reduces CMV RNA accumulation with 20-75% viral elimination rate	Kumar et al. (2016)
*Peanibacillus lentimorbus* B-30488	Tomato	*Scelerotium rolfsii*	Southern blight	Increased expression of PR genes but down-regulation of antioxidant enzymes and ethylene signaling	Reduces fungal dry biomass, mycelia growth	Dixit et al. (2016)
*Bacillus amyloliquefaciens* SQRT3	Tomato	*Ralstonia solanacearum*	Bacterial wilt	Modulates JA/ET/SA hormonal signaling and POD, PPO expression	Suppresses disease incidence with 84.1% biocontrol efficacy	Chunyu et al. (2017)
*Bacillus subtilis*	Wheat	*Zymoseptoria tritici*	Septoria tritici blotch	Produces lipopeptides such as mycosubtilin, surfactin, fengycin	Reduces mycelia growth	Mejri et al. (2018)
*Bacillus velezensis*	Pepper	*Botrytis cinerea*	Gray mold	Stimulates SA signaling genes and antioxidants (SOD, CAT and POD)	Suppresses sporulation, mycelia growth	Jiang et al. (2018)
*Pseudomonas aeruginosa*	Rapeseed	*Botrytis cinerea*	Gray mold	Activates MAPK and PR genes	Reduces pathogenic lesions size, mycelium development	Monnier et al. (2018)
*Bacillus velezensis* QST713	Button mushroom	*Trichoderma aggressivum* f*. europaeum*	Green mold	Produces antifungal secondary metabolites (macrolactin, bacillaene, bacillomycin D, fengycin, surfactin,bacilysin, subtilin- like/ericin, difficidin and bacillibactin (siderophore)	Inhibits fungus sporulation, mycelium growth	Pandin et al. (2018)
*Bacillus amyloliquefaciens* Ba13	Tomato	*Tomato yellow leaf curl virus*	–	Induces expression of resistance related genes and defense enzymes	Milder disease symptoms (stunting, yellowing, curling of leaves) with decrease in virus load in leaves	Guo et al. (2019)
*Bacillus velezensis* OEE1	Olive	*Verticillium dahliae*	Verticillium wilt	Produces antifungal secondary metabolites/lipopeptides (surfactin A, iturin C and D, bacillomycin C, fengycin A, B and D, plipastatin, macrolactin, bacillaene, difficidin and bacilysin)	Inhibits conidia, microsclerotia germination (92% inhibition)	Azabou et al. (2020)
*Bacillus velezensis* (C2)	Tomato	*Verticillium dahliae*	Verticillium wilt	Production of metabolites, lipopeptides and lytic enzymes	Significant reduction in disease incidence (70.43 ± 7.08%)	Dhouib et al. (2019)
*Bacillus amyloliquefaciens* YN201732	Tobacco	*Erysiphe cichoracearum*	Tobacco powdery mildew	Promotes the expression of PPO and chitinases, also triggers JA/ET signaling	Inhibits conidia germination (86.11%)	Jiao et al. (2020)
*Herbaspirillum seropedicae*	Micro-Tom Tomato	*Xanthomonas euvesicatoria*	Bacterial spot	Reduces the concentration of organic acids	50% reduction in disease severity	da Silva et al. (2021)

## Developing stress resilient crops

**Figure 2:**
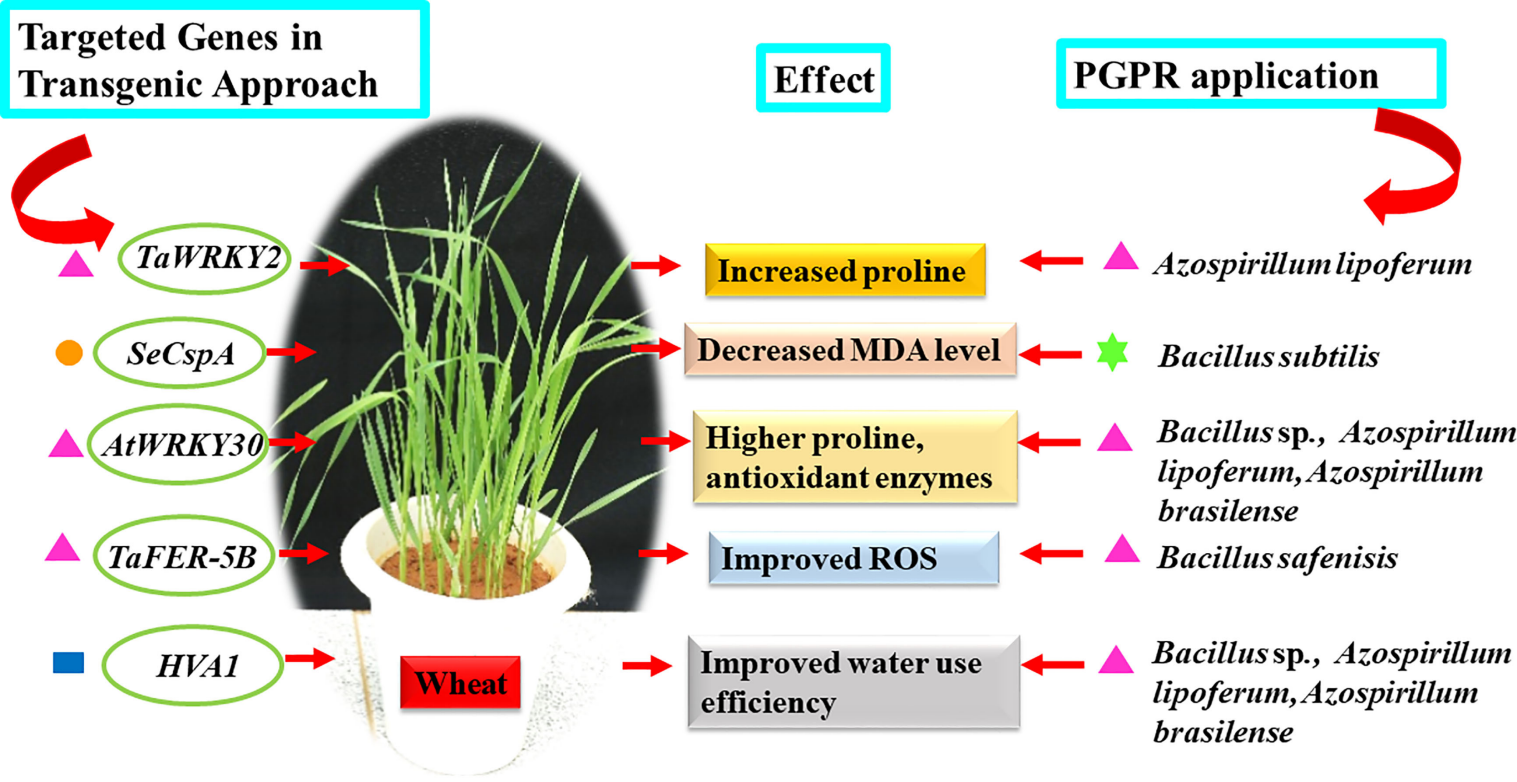
Model representing common physiological responses observed in wheat crop *via* transgenic approach and PGPRs application. Different colored symbols in the figure indicates the conditions of the experiment (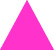
Pots, 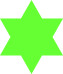
Filter Paper, 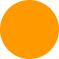
Hydroponics, 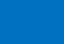
Greenhouse potting mix).

To full fill the goal of providing nutritious food for an increasing population, it is critical to develop resilient crops which can withstand the pressure of climate change. Scientists are strategizing, investigating, and discovering key genes which can aid in developing new transgenic crops tolerant to biotic and abiotic stresses without compromising on productivity. Wheat and rice are major staple crops grown across different regions of the world. Hence a lot of efforts are being made to develop better varieties through the classical breeding approach and newer biotechnological tools. The transgenic approach is one of the common techniques for inserting a gene of interest to achieve the desired trait. Among the transgenic approach, one of the studies illustrated that mutated transcription factor (*HaHB4*) from sunflower belonging to homeodomain-leucine zipper family (*HD-Zip*) improved water use efficiency and productivity of wheat (González et al., 2019). In another study introduction of beta gene-encoding choline dehydrogenase enhanced the glycine betaine content making the transformed wheat tolerant to drought (He et al., 2011). Similarly, in rice introduction of transcription factor *PeSNAC-1* lead to increased production of proline, thereby making the plant tolerant to salinity and drought (Hou et al., 2020). Also, insertion of gene *MYB49* lead to increased POD, SOD activity, chlorophyll content offering better resistance to drought, salinity and pathogen *Phytophthora infestans* in tomato (Cui et al., 2018). The transcription factor, *SlDREB3* increased membrane stability and prevented ROS from imparting tolerance to chilling (Wang et al., 2019). The above studies clearly suggest that modifications at the genetic level either by inserting specific genes; altering transcription factor families like *WRKY, DREB, MYB, NAC* and *ERF* and modifying signal transduction genes significantly enhances the tolerance of plants to various stresses (Khan et al., 2019d). Similar physiological changes can be introduced in target plants by applying appropriate PGPRs (**Tables 1**, **2** and **Figure 2**). In wheat, over expression of *TaWRKY2* (drought stress tolerance gene) improved drought tolerance by withholding water for 8-10 days before re-watering and enhancing proline content compared to wild type plant (Gao et al., 2018). Similarly, application of *Azospirillum lipoferum* significantly augmented the proline content in wheat seedling resulting in higher drought tolerance by withholding water for 10 days before watering (Kanwal et al., 2017). Engineering of *E. coli cold shock protein* (*CspA* and *CspB)* genes to convert it into plant-preferred codon namely *SeCspA* and *SeCspB* resulted in better stress tolerance potential by lowering MDA content, preventing water loss, reduced Na^+^ level and higher levels of chlorophyll, proline content under drought and salt stresses (200 mM NaCl) compared to the control wheat plants (Yu et al., 2017), similar response was evoked by endophytic strain *Bacillus subtilis* in wheat wherein lower MDA level was observed in plants grown under (340 mM NaCl) compared to control counter parts (Lastochkina et al., 2017). Cloning of *Arabidopsis WRKY30 (AtWRKY30)* transcription factor followed by its over-expression in wheat seedling subjected to drought stress by with-holding water for 12 days exhibited enhanced activities of antioxidant enzymes CAT, SOD, POX and APX (El-Esawi et al., 2019). Similar observation was made by (Akhtar et al., 2021) wherein antioxidant enzyme namely SOD, CAT and POX increased upon treating wheat with *Bacillus* sp., *Azospirillum lipoferum* and *Azospirillum brasilense* and subjected to drought stress by withholding water to 40% field capacity. Overexpression of *TaFER-5B* improved multiple stress including heat stress tolerance in wheat induced by keeping 10 days old seedling under 40°C, the protective mechanism was attributed to ROS scavenging activity (Zang et al., 2017). Similarly, modulation in expression of heat shock proteins (HSPs) was noticed in wheat seeds primed with *Bacillus safensis.* The treated plants tolerated heat shock (40°C) without generation of excessive ROS (Sarkar et al., 2021). Introduction of *HVA1* (ABA-responsive barley gene) in wheat improved water use efficiency subjected to drought stress in wheat (Sivamani et al., 2000). Similarly, application of bacteria based bioformulation improved relative water content and range of antioxidants of wheat seedlings experiencing drought stress (40% the field capacity) (Akhtar et al., 2021). Several scientific studies conclusively indicate that applications of PGPRs could lead to development of resilient plants tolerant to sudden fluctuations in the weather, rise in temperature, salinity, drought, disease and insect attack. The association between plant and rhizospheric soil microbes is a functionally dynamic association and changes in the environment are perceived even at miniscule level triggering a cascade of appropriate stress-related responses making the plant tolerant to perceived challenges. In spite of beneficial attribute of transgenic crops, their acceptability for human consumption is still an issue of concern. Hence, knowledge-based application of the right PGPR could be a sustainable and ecofriendly approach to develop stress resilient crops.

## Conclusion

A sustainable agriculture system strives to protect the environment, without compromising crop yield to provide sufficient food for the growing population. In order to provide adequate food for all, approaches like conventional breeding and genetic engineering have been extensively used for crop improvement. But these processes are costly, tedious, labor intensive and raise safety issues. Further, to improve crop productivity, heavy doses of chemical fertilizers, pesticides, and fungicides have been rampantly used which lead to large scale deterioration of the environment and soil health ultimately impacting human health. Hence, ecofriendly sustainable means are being explored to increase plant productivity and in this respect application of microbial based biostimulants in agriculture is one of the promising means. PGPR modulates physiological responses in crops and equips them to survive under abiotic and biotic stresses. The majority of these responses are common to both biotic and abiotic challenges implying that the impact of PGPR in plant systems is broad-based, interaction is multifarious and beneficial in more than one way. Further studies are necessary to unravel the underlying mechanism of plant-microbe interactions and to understand the signal transduction network in an integrated perspective. Holistic information about plant-PGPR functionality will pave the way for developing novel microbial based biostimulants for boosting crop yield for future generations in a sustainable manner.

## Author contributions

PK designed, drafted, revised, finalized the original manuscript and prepared the figures; NA drafted and revised the manuscript. Structuring, reviewing, and editing were done by SS. Conceptualization and input in hormonal interaction component was done by PP. Conceptualization, reviewing and editing was done by AP. All authors contributed to the article and approved the submitted version.

## References

[B1] Abbasi S. Sadeghi A. Safaie N. (2020). *Streptomyces* alleviate drought stress in tomato plants and modulate the expression of transcription factors ERF1 and WRKY70 genes. Sci. Hortic. 265, 109206. doi: 10.1016/j.scienta.2020.109206

[B2] Abbas R. Rasul S. Aslam K. Baber M. Shahid M. Mubeen F.. (2019). Halotolerant PGPR: A hope for cultivation of saline soils. J. King Saud Univ. Sci. 31, 1195–1201. doi: 10.1016/j.jksus.2019.02.019

[B3] Abd El-Daim I. A. Bejai S. Meijer J. (2014). Improved heat stress tolerance of wheat seedlings by bacterial seed treatment. Plant Soil 379, 337–350. doi: 10.1007/s11104-014-2063-3

[B4] Abd El-Daim I. A. Bejai S. Meijer J. (2019). *Bacillus velezensis* 5113 induced metabolic and molecular reprogramming during abiotic stress tolerance in wheat. Sci. Rep. 9, 16282. doi: 10.1038/s41598-019-52567-x 31704956 PMC6841942

[B5] Agarwal P. Singh P. C. Chaudhry V. Shirke P. A. Chakrabarty D. Farooqui A.. (2019). PGPR-induced *OsASR6* improves plant growth and yield by altering root auxin sensitivity and the xylem structure in transgenic *Arabidopsis thaliana* . J. Plant Physiol. 240, 153010. doi: 10.1016/j.jplph.2019.153010 31352021

[B6] Ahluwalia O. Singh P. C. Bhatia R. (2021). A review on drought stress in plants: Implications, mitigation and the role of plant growth promoting rhizobacteria. Resources Environ. Sustainability 5, 100032. doi: 10.1016/j.resenv.2021.100032

[B7] Ait Barka E. Nowak J. Clément C. (2006). Enhancement of chilling resistance of inoculated grapevine plantlets with a plant growth-promoting rhizobacterium, *Burkholderia phytofirmans* strain PsJN. Appl. Environ. Microbiol. 72, 7246–7252. doi: 10.1128/AEM.01047-06 16980419 PMC1636148

[B8] Akbar A. Han B. Khan A. H. Feng C. Ullah A. Khan A. S.. (2022). A transcriptomic study reveals salt stress alleviation in cotton plants upon salt tolerant PGPR inoculation. Environ. Exp. Bot. 200, 104928. doi: 10.1016/j.envexpbot.2022.104928

[B9] Akhtar N. Ilyas N. Mashwani Z. R. Hayat R. Yasmin H. Noureldeen A.. (2021). Synergistic effects of plant growth promoting rhizobacteria and silicon dioxide nano-particles for amelioration of drought stress in wheat. Plant Physiol. Biochem. 166, 160–176. doi: 10.1016/j.plaphy.2021.05.039 34116336

[B10] Ali B. Hafeez A. Ahmad S. Javed M. A. Afridi M. S. Dawoud T. M.. (2022). *Bacillus thuringiensis* PM25 ameliorates oxidative damage of salinity stress in maize *via* regulating growth, leaf pigments, antioxidant defense system, and stress responsive gene expression. Front. Plant Sci. 13. doi: 10.3389/fpls.2022.921668 PMC9366557 35968151

[B11] Ali S. Z. Sandhya V. Grover M. Kishore N. Rao L. V. Venkateswarlu B. (2009). *Pseudomonas* sp. strain AKM-P6 enhances tolerance of sorghum seedlings to elevated temperatures. Biol. Fertil. Soils 46, 45–55. doi: 10.1007/s00374-009-0404-9

[B12] Ali S. Z. Sandhya V. Grover M. Linga V. R. Bandi V. (2011). Effect of inoculation with a thermotolerant plant growth promoting *Pseudomonas putida* strain AKMP7 on growth of wheat (*Triticum* spp.) under heat stress. J. Plant Interact. 6, 239–246. doi: 10.1080/17429145.2010.545147

[B13] Amini S. Maali-Amiri R. Kazemi-Shahandashti S. S. López-Gómez M. Sadeghzadeh B. Sobhani-Najafabadi A.. (2021). Effect of cold stress on polyamine metabolism and antioxidant responses in chickpea. J. Plant Physiol. 258, 153387. doi: 10.1016/j.jplph.2021.153387 33636556

[B14] Ansari F. A. Ahmad I. Pichtel J. (2019). Growth stimulation and alleviation of salinity stress to wheat by the biofilm forming *Bacillus pumilus* strain FAB10. Appl. Soil Ecol. 143, 45–54. doi: 10.1016/j.apsoil.2019.05.023

[B15] Ansari F. A. Jabeen M. Ahmad I. (2021). *Pseudomonas azotoformans* FAP5, a novel biofilm-forming PGPR strain, alleviates drought stress in wheat plant. Int. J. Sci. Environ. Technol. 18, 3855–3870. doi: 10.1007/s13762-020-03045-9

[B16] Aremu A. O. Makhaye G. Tesfay S. Z. Gerrano A. S. Du Plooy C. P. Amoo S. O. (2022). Influence of commercial seaweed extract and microbial biostimulant on growth, yield, phytochemical content, and nutritional quality of five *Abelmoschus esculentus* genotypes. Agronomy 12, 428. doi: 10.3390/agronomy12020428

[B17] Arshad M. Shaharoona B. Mahmood T. (2008). Inoculation with *Pseudomonas* spp. containing ACC-deaminase partially eliminates the effects of drought stress on growth, yield, and ripening of pea (*Pisum sativum* l.). Pedosphere 18, 611–620. doi: 10.1016/S1002-0160(08)60055-7

[B18] Asghari B. Khademian R. Sedaghati B. (2020). Plant growth promoting rhizobacteria (PGPR) confer drought resistance and stimulate biosynthesis of secondary metabolites in pennyroyal (*Mentha pulegium* l.) under water shortage condition. Sci. Hortic. 263, 109132. doi: 10.1016/j.scienta.2019.109132

[B19] Ayaz M. Ali Q. Jiang Q. Wang R. Wang Z. Mu G.. (2022). Salt tolerant *Bacillus* strains improve plant growth traits and regulation of phytohormones in wheat under salinity stress. Plants 11, 2769. doi: 10.3390/plants11202769 36297795 PMC9608499

[B20] Ayed S. Bouhaouel I. Jebari H. Hamada W. (2022). Use of biostimulants: Towards sustainable approach to enhance durum wheat performances. Plants 11, 133. doi: 10.3390/plants11010133 35009136 PMC8747104

[B21] Azabou M. C. Gharbi Y. Medhioub I. Ennouri K. Barham H. Tounsi S.. (2020). The endophytic strain *Bacillus velezensis* OEE1: An efficient biocontrol agent against verticillium wilt of olive and a potential plant growth promoting bacteria. Biol. Control 142, 104168. doi: 10.1016/j.biocontrol.2019.104168

[B22] Backer R. Rokem J. S. Ilangumaran G. Lamont J. Praslickova D. Ricci E.. (2018). Plant growth-promoting rhizobacteria: Context, mechanisms of action, and roadmap to commercialization of biostimulants for sustainable agriculture. Front. Plant Sci. 9. doi: 10.3389/fpls.2018.01473 PMC6206271 30405652

[B23] Baltazar M. Correia S. Guinan K. J. Sujeeth N. Bragança R. Gonçalves B. (2021). Recent advances in the molecular effects of biostimulants in plants: An overview. Biomolecules 11, 1096. doi: 10.3390/biom11081096 34439763 PMC8394449

[B24] Bandeppa S. Paul S. Thakur J. K. Chandrashekar N. Umesh D. K. Aggarwal C.. (2019). Antioxidant, physiological and biochemical responses of drought susceptible and drought tolerant mustard (*Brassica juncea* l) genotypes to rhizobacterial inoculation under water deficit stress. Plant Physiol. Biochem. 143, 19–28. doi: 10.1016/j.plaphy.2019.08.018 31476528

[B25] Bano A. Fatima M. (2009). Salt tolerance in *Zea mays* (L). following inoculation with *Rhizobium* and *Pseudomonas* . Biol. Fertil. Soils 45, 405–413. doi: 10.1007/s00374-008-0344-9

[B26] Batool T. Ali S. Seleiman M. F. Naveed N. H. Ali A. Ahmed K.. (2020). Plant growth promoting rhizobacteria alleviates drought stress in potato in response to suppressive oxidative stress and antioxidant enzymes activities. Sci. Rep. 10, 1–19. doi: 10.1038/s41598-020-73489-z 33046721 PMC7550571

[B27] Baxter A. Mittler R. Suzuki N. (2014). ROS as key players in plant stress signalling. J. Exp. Bot. 65, 1229–1240. doi: 10.1093/jxb/ert375 24253197

[B28] Bharti N. Pandey S. S. Barnawal D. Patel V. K. Kalra A. (2016). Plant growth promoting rhizobacteria *Dietzia natronolimnaea* modulates the expression of stress responsive genes providing protection of wheat from salinity stress. Sci. Rep. 6, 34768. doi: 10.1038/srep34768 27708387 PMC5052518

[B29] Bhat M. A. Kumar V. Bhat M. A. Wani I. A. Dar F. L. Farooq I.. (2020). Mechanistic insights of the interaction of plant growth-promoting rhizobacteria (PGPR) with plant roots toward enhancing plant productivity by alleviating salinity stress. Front. Microbiol. 11 1952. doi: 10.3389/fmicb.2020.01952 PMC7468593 32973708

[B30] Brüggemann W. Beyel V. Brodka M. Poth H. Weil M. Stockhaus J. (1999). Antioxidants and antioxidative enzymes in wild-type and transgenic *Lycopersicon* genotypes of different chilling tolerance. Plant Sci. 140, 145–154. doi: 10.1016/S0168-9452(98)00220-9

[B31] Camejo D. Guzmán-Cedeño Á. Moreno A. (2016). Reactive oxygen species, essential molecules, during plant–pathogen interactions. Plant Physiol. Biochem. 103, 10–23. doi: 10.1016/j.plaphy.2016.02.035 26950921

[B32] Castiglione A. M. Mannino G. Contartese V. Bertea C. M. Ertani A. (2021). Microbial biostimulants as response to modern agriculture needs: Composition, role and application of these innovative products. Plants 10, 1533 doi: 10.3390/plants10081533 34451578 PMC8400793

[B33] Cataldo E. Fucile M. Mattii G. B. (2022). Biostimulants in viticulture: A sustainable approach against biotic and abiotic stresses. Plants 11, 162. doi: 10.3390/plants11020162 35050049 PMC8777853

[B34] Caulier S. Nannan C. Gillis A. Licciardi F. Bragard C. Mahillon J. (2019). Overview of the antimicrobial compounds produced by members of the *Bacillus subtilis* group. Front. Microbiol. 10. doi: 10.3389/fmicb.2019.00302 PMC6401651 30873135

[B35] Chakraborty U. Chakraborty B. Dey P. Chakraborty A. Sarkar J. (2019). Biochemical responses of wheat plants primed with *Ochrobactrum pseudogrignonense* and subjected to salinity stress. Agric. Res. 8, 427–440. doi: 10.1007/s40003-018-0394-7

[B36] Chandra D. Srivastava R. Glick B. R. Sharma A. K. (2018). Drought-tolerant *Pseudomonas* spp. improve the growth performance of finger millet (*Eleusine coracana* (L.) *Gaertn.*) under non-stressed and drought-stressed conditions. Pedosphere 28, 227–240. doi: 10.1016/S1002-0160(18)60013-X

[B37] Chiappero J. del Rosario Cappellari L. Alderete L. G. S. Palermo T. B. Banchio E. (2019). Plant growth promoting rhizobacteria improve the antioxidant status in *Mentha piperita* grown under drought stress leading to an enhancement of plant growth and total phenolic content. Ind. Crop Prod. 139, 111553. doi: 10.1016/j.indcrop.2019.111553

[B38] Cho S. Park J. Han S. Anderson A. Yang K.-Y. Gardener B.. (2011). Identification and transcriptional analysis of priming genes in *Arabidopsis thaliana* induced by root colonization with *Pseudomonas chlororaphis* O6. Plant Pathol. J. 27, 272–279. doi: 10.5423/PPJ.2011.27.3.272

[B39] Choudhary M. Chandra P. Dixit B. Nehra V. Choudhary U. Choudhary S. (2022). Plant growth-promoting microbes: Role and prospective in amelioration of salt stress. Commun. Soil Sci. Plant Anal. 53, 1692–1711. doi: 10.1080/00103624.2022.2063316

[B40] Chunyu L. I. Weicong H. U. Bin P. A. N. Yan L. I. U. Saifei Y. U. A. N. Yuanyuan D. I. N. G.. (2017). Rhizobacterium *Bacillus amyloliquefaciens* strain SQRT3-mediated induced systemic resistance controls bacterial wilt of tomato. Pedosphere 27, 1135–1146. doi: 10.1016/S1002-0160(17)60406-5

[B41] Chu T. N. Tran B. T. H. Van Bui L. Hoang M. T. T. (2019). Plant growth-promoting rhizobacterium *Pseudomonas* PS01 induces salt tolerance in *Arabidopsis thaliana* . BMC Res. Notes 12, 1–7. doi: 10.1186/s13104-019-4046-1 30635071 PMC6330407

[B42] Colla G. Rouphael Y. (2020). Microalgae: New source of plant biostimulants. Agronomy 10, 1240 doi: 10.3390/agronomy10091240

[B43] Conrath U. Beckers G. J. Langenbach C. J. Jaskiewicz M. R. (2015). Priming for enhanced defense. Annu. Rev. Phytopathol. 53, 97–119. doi: 10.1146/annurev-phyto-080614-120132 26070330

[B44] Cruz de Carvalho M. H. (2008). Drought stress and reactive oxygen species: production, scavenging and signaling. Plant Signal. Behav. 3, 156–165. doi: 10.4161/psb.3.3.5536 19513210 PMC2634109

[B45] Cui J. Jiang N. Zhou X. Hou X. Yang G. Meng J.. (2018). Tomato MYB49 enhances resistance to *Phytophthora infestans* and tolerance to water deficit and salt stress. Planta 248, 1487–1503. doi: 10.1007/s00425-018-2987-6 30132153

[B46] Curá J. A. Franz D. R. Filosofía J. E. Balestrasse K. B. Burgueño L. E. (2017). Inoculation with *Azospirillum* sp. and *Herbaspirillum* sp. bacteria increases the tolerance of maize to drought stress. Microorganisms 5, 41. doi: 10.3390/microorganisms5030041 28933739 PMC5620632

[B47] Danish S. Zafar-Ul-Hye M. Hussain S. Riaz M. Qayyum M. F. (2020). Mitigation of drought stress in maize through inoculation with drought tolerant ACC deaminase containing PGPR under axenic conditions. Pak. J. Bot. 52, 49–60. doi: 10.30848/PJB2020-1(7)

[B48] da Silva A. P. S. Olivares F. L. Sudré C. P. Peres L. E. P. Canellas N. A. da Silva R. M.. (2021). Attenuations of bacterial spot disease *Xanthomonas euvesicatoria* on tomato plants treated with biostimulants. Chem. Biol. Technol. Agric. 8, 1–9. doi: 10.1186/s40538-021-00240-9

[B49] Desoky E. S. M. Saad A. M. El-Saadony M. T. Merwad A. R. M. Rady M. M. (2020). Plant growth-promoting rhizobacteria: Potential improvement in antioxidant defense system and suppression of oxidative stress for alleviating salinity stress in *Triticum aestivum* (L.) plants. Biocatal. Agric. Biotechnol. 30, 101878. doi: 10.1016/j.bcab.2020.101878

[B50] Dhouib H. Zouari I. Abdallah D. B. Belbahri L. Taktak W. Triki M. A.. (2019). Potential of a novel endophytic *Bacillus velezensis* in tomato growth promotion and protection against verticillium wilt disease. Biol. Control 139, 104092. doi: 10.1016/j.biocontrol.2019.104092

[B51] Dimopoulou A. Theologidis I. Benaki D. Koukounia M. Zervakou A. Tzima A.. (2021). Direct antibiotic activity of bacillibactin broadens the biocontrol range of *Bacillus amyloliquefaciens* MBI600. Msphere 6, e00376–e00321. doi: 10.1128/mSphere.00376-21 34378986 PMC8386435

[B52] Dixit R. Agrawal L. Gupta S. Kumar M. Yadav S. Chauhan P. S.. (2016). Southern blight disease of tomato control by 1-aminocyclopropane-1-carboxylate (ACC) deaminase producing *Paenibacillus lentimorbus* b-30488. Plant Signal. Behav. 11, e1113363. doi: 10.1080/15592324.2015.1113363 26825539 PMC4883846

[B53] Dreesen F. E. De Boeck H. J. Janssens I. A. Nijs I. (2012). Summer heat and drought extremes trigger unexpected changes in productivity of a temperate annual/biannual plant community. Environ. Exp. Bot. 79, 21–30. doi: 10.1016/j.envexpbot.2012.01.005

[B54] Drobek M. Frąc M. Cybulska J. (2019). Plant biostimulants: Importance of the quality and yield of horticultural crops and the improvement of plant tolerance to abiotic stress–a review. Agronomy 9, 335. doi: 10.3390/agronomy9060335

[B55] Ebrahimi S. Eini O. Koolivand D. (2020). Arbuscular mycorrhizal symbiosis enhances virus accumulation and attenuates resistance-related gene expression in tomato plants infected with *Beet curly top Iran virus* . J. Plant Dis. Prot. 127, 341–348. doi: 10.1007/s41348-020-00299-w

[B56] Egamberdieva D. Davranov K. Wirth S. Hashem A. Abd Allah E. F. (2017). Impact of soil salinity on the plant-growth - promoting and biological control abilities of root associated bacteria. Saudi J. Biol. Sci. 24, 1601–1608. doi: 10.1016/j.sjbs.2017.07.004 29062259 PMC5643845

[B57] El-Esawi M. A. Al-Ghamdi A. A. Ali H. M. Ahmad M. (2019). Overexpression of *AtWRKY30* transcription factor enhances heat and drought stress tolerance in wheat (*Triticum aestivum* l.). Genes 10, E163. doi: 10.3390/genes10020163 PMC6410048 30791662

[B58] Eom S. H. Ahn M. A. Kim E. Lee H. J. Lee J. H. Wi S. H.. (2022). Plant response to cold stress: Cold stress changes antioxidant metabolism in heading type kimchi cabbage (*Brassica rapa* l. ssp. pekinensis). Antioxidants 11, 700. doi: 10.3390/antiox11040700 35453385 PMC9031148

[B59] Erice G. Ruíz-Lozano J. M. Zamarreño Á.M. García-Mina J. M. Aroca R. (2017). Transcriptomic analysis reveals the importance of JA-ile turnover in the response of *Arabidopsis* plants to plant growth promoting rhizobacteria and salinity. Environ. Exp. Bot. 143, 10–19. doi: 10.1016/j.envexpbot.2017.08.006

[B60] Expósito C. D. V. López J.Á. Liu J. Bao N. Liang J. Zhang J. (2022). Development of a cold-active microbial compound biofertilizer on the improvement for rice (*oryza sativa* l.) tolerance at low-temperature. Rhizosphere 24, 100586. doi: 10.1016/j.rhisph.2022.100586

[B61] Fadiji A. E. Babalola O. O. Santoyo G. Perazzolli M. (2022). The potential role of microbial biostimulants in the amelioration of climate change-associated abiotic stresses on crops. Front. Microbiol. 12. doi: 10.3389/fmicb.2021.829099 PMC8795815 35095828

[B62] Franzoni G. Cocetta G. Prinsi B. Ferrante A. Espen L. (2022). Biostimulants on crops: Their impact under abiotic stress conditions. Horticulturae 8, 189. doi: 10.3390/horticulturae8030189

[B63] Fu H. Z. Marian M. Enomoto T. Hieno A. Ina H. Suga H.. (2020). Biocontrol of tomato bacterial wilt by foliar spray application of a novel strain of *endophytic bacillus* sp. Microbes Environ. 35, ME20078. doi: 10.1264/jsme2.ME20078 33012743 PMC7734409

[B64] Gamalero E. Glick B. R. (2022). Recent advances in bacterial amelioration of plant drought and salt stress. Biology 11, 437. doi: 10.3390/biology11030437 35336811 PMC8945159

[B65] Gamez R. Cardinale M. Montes M. Ramirez S. Schnell S. Rodriguez F. (2019). Screening, plant growth promotion and root colonization pattern of two rhizobacteria (*Pseudomonas fluorescens* Ps006 and *Bacillus amyloliquefaciens* Bs006) on banana cv. williams (*Musa acuminata* colla). Microbiol. Res. 220, 12–20. doi: 10.1016/j.micres.2018.11.006 30744815

[B66] Gao H. Wang Y. Xu P. Zhang Z. (2018). Overexpression of a WRKY transcription factor *TaWRKY2* enhances drought stress tolerance in transgenic wheat. Front. Plant Sci. 9. doi: 10.3389/fpls.2018.00997 PMC6090177 30131813

[B67] Ge H. Zhang F. (2019). Growth-promoting ability of *Rhodopseudomonas palustris* G5 and its effect on induced resistance in cucumber against salt stress. J. Plant Growth Regul. 38, 180–188. doi: 10.1007/s00344-018-9825-8

[B68] González F. G. Capella M. Ribichich K. F. Curín F. Giacomelli J. I. Ayala F.. (2019). Field-grown transgenic wheat expressing the sunflower gene *HaHB4* significantly outyields the wild type. J. Exp. Bot. 70, 1669–1681. doi: 10.1093/jxb/erz037 30726944 PMC6411379

[B69] Guo Q. Li Y. Lou Y. Shi M. Jiang Y. Zhou J.. (2019). *Bacillus amyloliquefaciens* Ba13 induces plant systemic resistance and improves rhizosphere microecology against tomato yellow leaf curl virus disease. Appl. Soil Ecol. 137, 154–166. doi: 10.1016/j.apsoil.2019.01.015

[B70] Guo Q. Li X. Niu L. Jameson P. E. Zhou W. (2021). Transcription-associated metabolomic adjustments in maize occur during combined drought and cold stress. Plant Physiol. 186, 677–695. doi: 10.1093/plphys/kiab050 33582802 PMC8154062

[B71] Gupta S. Pandey S. (2019). ACC deaminase producing bacteria with multifarious plant growth promoting traits alleviates salinity stress in French bean (*Phaseolus vulgaris*) plants. Front. Microbiol. 10. doi: 10.3389/fmicb.2019.01506 PMC6629829 31338077

[B72] Gururani M. A. Upadhyaya C. P. Baskar V. Venkatesh J. Nookaraju A. Park S. W. (2013). Plant growth-promoting rhizobacteria enhance abiotic stress tolerance in *Solanum tuberosum* through inducing changes in the expression of ROS-scavenging enzymes and improved photosynthetic performance. J. Plant Growth Regul. 32, 245–258. doi: 10.1007/s00344-012-9292-6

[B73] Habib S. H. Kausar H. Saud H. M. (2016). Plant growth-promoting rhizobacteria enhance salinity stress tolerance in okra through ROS-scavenging enzymes. BioMed. Res. Int. 2016, e6284547. doi: 10.1155/2016/6284547 PMC4756578 26951880

[B74] Hamid B. Zaman M. Farooq S. Fatima S. Sayyed R. Z. Baba Z. A.. (2021). Bacterial plant biostimulants: A sustainable way towards improving growth, productivity, and health of crops. Sustain 13, 2856. doi: 10.3390/su13052856

[B75] Hayat S. Hayat Q. Alyemeni M. N. Wani A. S. Pichtel J. Ahmad A. (2012). Role of proline under changing environments: A review. Plant Signal. Behav. 7, 1456–1466. doi: 10.4161/psb.21949 22951402 PMC3548871

[B76] Hendriksen N. B. (2022). Microbial biostimulants–the need for clarification in EU regulation. Trends Microbiol. 30, 311–313. doi: 10.1016/j.tim.2022.01.008 35125255

[B77] He A. Niu S. Yang D. Ren W. Zhao L. Sun Y.. (2021). Two PGPR strains from the rhizosphere of *Haloxylon ammodendron* promoted growth and enhanced drought tolerance of ryegrass. Plant Physiol. Biochem. 161, 74–85. doi: 10.1016/j.plaphy.2021.02.003 33578287

[B78] He C. Zhang W. Gao Q. Yang A. Hu X. Zhang J. (2011). Enhancement of drought resistance and biomass by increasing the amount of glycine betaine in wheat seedlings. Euphytica 177, 151–167. doi: 10.1007/s10681-010-0263-3

[B79] Hou D. Zhao Z. Hu Q. Li L. Vasupalli N. Zhuo J.. (2020). PeSNAC-1 a NAC transcription factor from moso bamboo (*Phyllostachys edulis*) confers tolerance to salinity and drought stress in transgenic rice. Tree Physiol. 40, 1792–1806. doi: 10.1093/treephys/tpaa099 32761243

[B80] Hwarari D. Guan Y. Ahmad B. Movahedi A. Min T. Hao Z.. (2022). ICE-CBF-COR signaling cascade and its regulation in plants responding to cold stress. Int. J. Mol. Sci. 23, 1549. doi: 10.3390/ijms23031549 35163471 PMC8835792

[B81] Irshad A. Rehman R. N. U. Kareem H. A. Yang P. Hu T. (2021). Addressing the challenge of cold stress resilience with the synergistic effect of *Rhizobium* inoculation and exogenous melatonin application in *Medicago truncatula* . Ecotoxicol. Environ. Saf. 226, 112816. doi: 10.1016/j.ecoenv.2021.112816 34597844

[B82] Issa A. Esmaeel Q. Sanchez L. Courteaux B. Guise J.-F. Gibon Y.. (2018). Impacts of *Paraburkholderia phytofirmans* strain PsJN on tomato (*Lycopersicon esculentum* l.) under high temperature. Front. Plant Sci. 9. doi: 10.3389/fpls.2018.01397 PMC6201190 30405648

[B83] Jatan R. Chauhan P. S. Lata C. (2019). *Pseudomonas putida* modulates the expression of miRNAs and their target genes in response to drought and salt stresses in chickpea (*Cicer arietinum* l.). Genomics 111c, 509–519. doi: 10.1016/j.ygeno.2018.01.007 29331610

[B84] Jiang C. H. Liao M. J. Wang H. K. Zheng M. Z. Xu J. J. Guo J. H. (2018). *Bacillus velezensis*, a potential and efficient biocontrol agent in control of pepper gray mold caused by *Botrytis cinerea* . Biol. Control 126, 147–157. doi: 10.1016/j.biocontrol.2018.07.017

[B85] Jiao R. Munir S. He P. Yang H. Wu Y. Wang J.. (2020). Biocontrol potential of the endophytic *Bacillus amyloliquefaciens* YN201732 against tobacco powdery mildew and its growth promotion. Biol. Control 143, 104160. doi: 10.1016/j.biocontrol.2019.104160

[B86] Jin N. Jin L. Wang S. Li J. Liu F. Liu Z.. (2022). Reduced chemical fertilizer combined with bio-organic fertilizer affects the soil microbial community and yield and quality of lettuce. Front. Microbiol. 13. doi: 10.3389/fmicb.2022.863325 PMC9069001 35531292

[B87] Ji J. Yuan D. Jin C. Wang G. Li X. Guan C. (2020). Enhancement of growth and salt tolerance of rice seedlings (*Oryza sativa* l.) by regulating ethylene production with a novel halotolerant PGPR strain *Glutamicibacter* sp. YD01 containing ACC deaminase activity. Acta Physiol. Plant 42, 1–17. doi: 10.1007/s11738-020-3034-3

[B88] Ji C. Zhang M. Kong Z. Chen X. Wang X. Ding W.. (2021). Genomic analysis reveals potential mechanisms underlying promotion of tomato plant growth and antagonism of soilborne pathogens by *Bacillus amyloliquefaciens* Ba13. Microbiol. Spectr. 9, e01615–e01621. doi: 10.1128/Spectrum.01615-21 34756081 PMC8579842

[B89] Kang S. M. Khan A. L. Waqas M. Asaf S. Lee K. E. Park Y. G. (2019a). Integrated phytohormone production by the plant growth-promoting rhizobacterium *Bacillus tequilensis* SSB07 induced thermotolerance in soybean. J. Plant Interact. 14, 416–423. doi: 10.1080/17429145.2019.1640294

[B90] Kang S. M. Shahzad R. Bilal S. Khan A. L. Park Y. G. Lee K. E.. (2019b). Indole-3-acetic-acid and ACC deaminase producing *Leclercia adecarboxylata* MO1 improves *Solanum lycopersicum* l. growth and salinity stress tolerance by endogenous secondary metabolites regulation. BMC Microbiol. 19, 1–14. doi: 10.1186/s12866-019-1450-6 31023221 PMC6485084

[B91] Kanwal S. Ilyas N. Batool N. Arshad M. (2017). Amelioration of drought stress in wheat by combined application of PGPR, compost, and mineral fertilizer. J. Plant Nutr. 40, 1250–1260. doi: 10.1080/01904167.2016.1263322

[B92] Kaya C. Şenbayram M. Akram N. A. Ashraf M. Alyemeni M. N. Ahmad P. (2020). Sulfur-enriched leonardite and humic acid soil amendments enhance tolerance to drought and phosphorus deficiency stress in maize (*Zea mays* l.). Sci. Rep. 10, 1–13. doi: 10.1038/s41598-020-62669-6 32286357 PMC7156716

[B93] Kazemi-Shahandashti S. S. Maali-Amiri R. (2018). Global insights of protein responses to cold stress in plants: Signaling, defence, and degradation. J. Plant Physiol. 226, 123–135. doi: 10.1016/j.jplph.2018.03.022 29758377

[B94] Kazerooni E. A. Maharachchikumbura S. S. Adhikari A. Al-Sadi A. M. Kang S. M. Kim L. R.. (2021). Rhizospheric *Bacillus amyloliquefaciens* protects *Capsicum annuum* cv. geumsugangsan from multiple abiotic stresses *via* multifarious plant growth-promoting attributes. Front. Plant Sci. 12. doi: 10.3389/fpls.2021.669693 PMC8185346 34113368

[B95] Khan S. Anwar S. Yu S. Sun M. Yang Z. Gao Z. Q. (2019d). Development of drought-tolerant transgenic wheat: Achievements and limitations. Int. J. Mol. Sci. 20, 3350. doi: 10.3390/ijms20133350 31288392 PMC6651533

[B96] Khan M. A. Asaf S. Khan A. L. Adhikari A. Jan R. Ali S.. (2019b). Halotolerant rhizobacterial strains mitigate the adverse effects of NaCl stress in soybean seedlings. BioMed. Res. Int. 2019, 9530963. doi: 10.1155/2019/9530963 31886270 PMC6925695

[B97] Khan M. A. Asaf S. Khan A. L. Jan R. Kang S. M. Kim K. M.. (2020). Thermotolerance effect of plant growth-promoting *Bacillus cereus* SA1 on soybean during heat stress. BMC Microbiol. 20, 175. doi: 10.1186/s12866-020-01822-7 32571217 PMC7310250

[B98] Khan M. A. Asaf S. Khan A. L. Ullah I. Ali S. Kang S. M.. (2019c). Alleviation of salt stress response in soybean plants with the endophytic bacterial isolate *curtobacterium* sp. SAK1. Ann. Microbiol. 69, 797–808. doi: 10.1007/s13213-019-01470-x

[B99] Khan M. I. Poor P. Janda T. (2022). Salicylic acid: A versatile signaling molecule in plants. J. Plant Growth Regul. 41, 1887–1890. doi: 10.1007/s00344-022-10692-4

[B100] Khan A. Uddin S. Zhao X. Q. Javed M. T. Khan K. S. Bano A.. (2016). *Bacillus pumilus* enhances tolerance in rice (*Oryza sativa* l.) to combined stresses of NaCl and high boron due to limited uptake of na^+^ . Environ. Exp. Bot. 124, 120–129. doi: 10.1016/j.envexpbot.2015.12.011

[B101] Khan M. A. Ullah I. Waqas M. Hamayun M. Khan A. L. Asaf S.. (2019a). Halo-tolerant rhizospheric *Arthrobacter woluwensis* AK1 mitigates salt stress and induces physio-hormonal changes and expression of *GmST1* and *GmLAX3* in soybean. Symbiosis 77, 9–21. doi: 10.1007/s13199-018-0562-3

[B102] Kim K. Jang Y. J. Lee S.-M. Oh B. T. Chae J. C. Lee K. J. (2014). Alleviation of salt stress by *Enterobacter* sp. EJ01 in tomato and *Arabidopsis* is accompanied by up-regulation of conserved salinity responsive factors in plants. Mol. Cells 37, 109–117. doi: 10.14348/molcells.2014.2239 24598995 PMC3935623

[B103] Kim S. T. Yoo S. J. Weon H. Y. Song J. Sang M. K. (2022). *Bacillus butanolivorans* KJ40 contributes alleviation of drought stress in pepper plants by modulating antioxidant and polyphenolic compounds. Sci. Hortic. 301, 111111. doi: 10.1016/j.scienta.2022.111111

[B104] Kishor P. K. Sangam S. Amrutha R. N. Laxmi P. S. Naidu K. R. Rao K. S.. (2005). Regulation of proline biosynthesis, degradation, uptake and transport in higher plants: Its implications in plant growth and abiotic stress tolerance. Curr. Sci. 88, 424–438.

[B105] Kosar F. Akram N. A. Ashraf M. Ahmad A. Alyemeni M. N. Ahmad P. (2021). Impact of exogenously applied trehalose on leaf biochemistry, achene yield and oil composition of sunflower under drought stress. Physiol. Plant 172, 317–333. doi: 10.1111/ppl.13155 32562257

[B106] Kour D. Yadav A. N. (2022). Bacterial mitigation of drought stress in plants: Current perspectives and future challenges. Curr. Microbiol. 79, 1–19. doi: 10.1007/s00284-022-02939-w 35834053

[B107] Kumar S. Chauhan P. S. Agrawal L. Raj R. Srivastava A. Gupta S.. (2016). *Paenibacillus lentimorbus* inoculation enhances tobacco growth and extenuates the virulence of *Cucumber mosaic virus* . PLos One 11, e0149980. doi: 10.1371/journal.pone.0149980 26934600 PMC4774868

[B108] Kumar A. Patel J. S. Meena V. S. Srivastava R. (2019). Recent advances of PGPR based approaches for stress tolerance in plants for sustainable agriculture. Biocatal. Agric. Biotechnol. 20, 101271. doi: 10.1016/j.bcab.2019.101271

[B109] Kumawat K. C. Nagpal S. Sharma P. (2022). Potential of plant growth-promoting rhizobacteria-plant interactions in mitigating salt stress for sustainable agriculture: A review. Pedosphere 32, 223–245. doi: 10.1016/S1002-0160(21)60070-X

[B110] Lastochkina O. Pusenkova L. Yuldashev R. Babaev M. Garipova S. Blagova D.. (2017). Effects of *Bacillus subtilis* on some physiological and biochemical parameters of *Triticum aestivum* l. (wheat) under salinity. Plant Physiol. Biochem. 121, 80–88. doi: 10.1016/j.plaphy.2017.10.020 29096176

[B111] Laxa M. Liebthal M. Telman W. Chibani K. Dietz K. J. (2019). The role of the plant antioxidant system in drought tolerance. Antioxidants 8, 94. doi: 10.3390/antiox8040094 30965652 PMC6523806

[B112] Lephatsi M. M. Meyer V. Piater L. A. Dubery I. A. Tugizimana F. (2021). Plant responses to abiotic stresses and rhizobacterial biostimulants: Metabolomics and epigenetics perspectives. Metabolites 11, 457. doi: 10.3390/metabo11070457 34357351 PMC8305699

[B113] Li H. Ding X. Wang C. Ke H. Wu Z. WANG Y.. (2016). Control of tomato yellow leaf curl virus disease by *Enterobacter asburiae*BQ9 as a result of priming plant resistance in tomatoes. Turkish J. Biol. 40, 150–159. doi: 10.3906/biy-1502-12

[B114] Lim J. H. Kim S. D. (2013). Induction of drought stress resistance by multi-functional PGPR *Bacillus licheniformis* K11 in pepper. Plant Pathol. J. 29, 201–208. doi: 10.5423/PPJ.SI.02.2013.0021 25288947 PMC4174774

[B115] Lin Y. Jones M. L. (2022). Evaluating the growth-promoting effects of microbial biostimulants on greenhouse floriculture crops. HortScience 57, 97–109. doi: 10.21273/HORTSCI16149-21

[B116] Lipiec J. Doussan C. Nosalewicz A. Kondracka K. (2013). Effect of drought and heat stresses on plant growth and yield: A review. Int. Agrophys. 27, 463–477. doi: 10.2478/intag-2013-0017

[B117] Li X. Sun P. Zhang Y. Jin C. Guan C. (2020). A novel PGPR strain *Kocuria rhizophila* Y1 enhances salt stress tolerance in maize by regulating phytohormone levels, nutrient acquisition, redox potential, ion homeostasis, photosynthetic capacity and stress-responsive genes expression. Environ. Exp. Bot. 174, 104023. doi: 10.1016/j.envexpbot.2020.104023

[B118] Liu S. Hao H. Lu X. Zhao X. Wang Y. Zhang Y.. (2017). Transcriptome profiling of genes involved in induced systemic salt tolerance conferred by *Bacillus amyloliquefaciens* FZB42 in *Arabidopsis thaliana* . Sci. Rep. 7, 1–13. doi: 10.1038/s41598-017-11308-8 28904348 PMC5597682

[B119] Liu W. Sikora E. Park S. W. (2020). Plant growth-promoting rhizobacterium, *Paenibacillus polymyxa* CR1, upregulates dehydration-responsive genes, *RD29A* and *RD29B*, during priming drought tolerance in arabidopsis. Plant Physiol. Biochem. 156, 146–154. doi: 10.1016/j.plaphy.2020.08.049 32947123

[B120] Luo L. Zhao C. Wang E. Raza A. Yin C. (2022). *Bacillus amyloliquefaciens* as an excellent agent for biofertilizer and biocontrol in agriculture: An overview for its mechanisms. Microbiol. Res. 259, 127016. doi: 10.1016/j.micres.2022.127016 35390741

[B121] Mansoor S. Kour N. Manhas S. Zahid S. Wani O. A. Sharma V.. (2021). Biochar as a tool for effective management of drought and heavy metal toxicity. Chemosphere 271, 129458. doi: 10.1016/j.chemosphere.2020.129458 33421912

[B122] Ma J. Peng J. Tian S. He Y. Zhang C. Jia N.. (2022). *Streptomyces* sp. TOR3209 alleviates cold stress in tomato plants. N. Z. J. Crop Hortic. Sci., 1–21. doi: 10.1080/01140671.2022.2066141

[B123] Meena M. Yadav G. Sonigra P. Nagda A. Mehta T. Swapnil P.. (2022). Role of elicitors to initiate the induction of systemic resistance in plants to biotic stress. Plant Stress 5, 100103. doi: 10.1016/j.stress.2022.100103

[B124] Mejri S. Siah A. Coutte F. Magnin-Robert M. Randoux B. Tisserant B.. (2018). Biocontrol of the wheat pathogen *Zymoseptoria tritici* using cyclic lipopeptides from *Bacillus subtilis* . Environ. Sci. pollut. Res. 25, 29822–29833. doi: 10.1007/s11356-017-9241-9 28634804

[B125] Mekureyaw M. F. Pandey C. Hennessy R. C. Nicolaisen M. H. Liu F. Nybroe O.. (2022). The cytokinin-producing plant beneficial bacterium *Pseudomonas fluorescens* G20-18 primes tomato (*Solanum lycopersicum*) for enhanced drought stress responses. J. Plant Physiol. 270, 153629. doi: 10.1016/j.jplph.2022.153629 35151004

[B126] Miceli A. Moncada A. Vetrano F. (2021). Use of microbial biostimulants to increase the salinity tolerance of vegetable transplants. Agronomy 11, 1143. doi: 10.3390/agronomy11061143

[B127] Mishra P. K. Bisht S. C. Ruwari P. Selvakumar G. Joshi G. K. Bisht J. K.. (2011). Alleviation of cold stress in inoculated wheat (*Triticum aestivum* l.) seedlings with psychrotolerant *Pseudomonads* from NW Himalayas. Arch. Microbiol. 193, 497–513. doi: 10.1007/s00203-011-0693-x 21442319

[B128] Mitra D. Rodriguez A. M. D. Cota F. I. P. Khoshru B. Panneerselvam P. Moradi S.. (2021). Amelioration of thermal stress in crops by plant growth-promoting rhizobacteria. Physiol. Mol. Plant Pathol. 115, 101679. doi: 10.1016/j.pmpp.2021.101679

[B129] Mokabel S. Olama Z. Ali S. El-Dakak R. (2022). The role of plant growth promoting rhizosphere microbiome as alternative biofertilizer in boosting *Solanum melongena* l. adaptation to salinity stress. Plants 11, 659. doi: 10.3390/plants11050659 35270129 PMC8912713

[B130] Monnier N. Furlan A. Botcazon C. Dahi A. Mongélard G. Cordelier S.. (2018). Rhamnolipids from *Pseudomonas aeruginosa* are elicitors triggering *Brassica napus* protection against *Botrytis cinerea* without physiological disorders. Front. Plant Sci. 9. doi: 10.3389/fpls.2018.01170 PMC6092566 30135699

[B131] Monteiro E. Gonçalves B. Cortez I. Castro I. (2022). The role of biostimulants as alleviators of biotic and abiotic stresses in grapevine: A review. Plants 11, 396. doi: 10.3390/plants11030396 35161376 PMC8839214

[B132] Moreno-Galván A. E. Cortés-Patiño S. Romero-Perdomo F. Uribe-Vélez D. Bashan Y. Bonilla R. R. (2020). Proline accumulation and glutathione reductase activity induced by drought-tolerant rhizobacteria as potential mechanisms to alleviate drought stress in Guinea grass. Appl. Soil Ecol. 147, 103367. doi: 10.1016/j.apsoil.2019.103367

[B133] Mukhtar T. Rehman S. U. Smith D. Sultan T. Seleiman M. Alsadon A.. (2020). Mitigation of heat stress in *Solanum lycopersicum* l. by ACC-deaminase and exopolysaccharide producing *Bacillus cereus*: Effects on biochemical profiling. Sustain 12, 2159. doi: 10.3390/su12062159

[B134] Murali M. Gowtham H. G. Singh S. B. Shilpa N. Aiyaz M. Niranjana S. R.. (2021a). Bio-prospecting of ACC deaminase producing rhizobacteria towards sustainable agriculture: A special emphasis on abiotic stress in plants. Appl. Soil Ecol. 168, 104142. doi: 10.1016/j.apsoil.2021.104142

[B135] Murali M. Singh S. B. Gowtham H. G. Shilpa N. Prasad M. Aiyaz M.. (2021b). Induction of drought tolerance in *Pennisetum glaucum* by ACC deaminase producing PGPR-*Bacillus amyloliquefaciens* through antioxidant defense system. Microbiol. Res. 253, 126891. doi: 10.1016/j.micres.2021.126891 34656832

[B136] Naseem H. Bano A. (2014). Role of plant growth-promoting rhizobacteria and their exopolysaccharide in drought tolerance of maize. J. Plant Interact. 9, 689–701. doi: 10.1080/17429145.2014.902125

[B137] Noor R. Yasmin H. Ilyas N. Nosheen A. Hassan M. N. Mumtaz S.. (2022). Comparative analysis of iron oxide nanoparticles synthesized from ginger (*Zingiber officinale*) and cumin seeds (*Cuminum cyminum*) to induce resistance in wheat against drought stress. Chemosphere 292, 133201. doi: 10.1016/j.chemosphere.2021.133201 34921860

[B138] Notununu I. Moleleki L. Roopnarain A. Adeleke R. (2022). Effects of plant growth-promoting rhizobacteria on the molecular responses of maize under drought and heat stresses: A review. Pedosphere 32, 90–106. doi: 10.1016/S1002-0160(21)60051-6

[B139] Olowe O. M. Akanmu A. O. Asemoloye M. D. (2020). Exploration of microbial stimulants for induction of systemic resistance in plant disease management. Ann. Appl. Biol. 177, 282–293. doi: 10.1111/aab.12631

[B140] Orozco-Mosqueda M. del C. Glick B. R. Santoyo G. (2020). ACC deaminase in plant growth-promoting bacteria (PGPB): An efficient mechanism to counter salt stress in crops. Microbiol. Res. 235, 126439. doi: 10.1016/j.micres.2020.126439 32097862

[B141] Othibeng K. Nephali L. Myoli A. Buthelezi N. Jonker W. Huyser J.. (2022). Metabolic circuits in sap extracts reflect the effects of a microbial biostimulant on maize metabolism under drought conditions. Plants 11, 510. doi: 10.3390/plants11040510 35214843 PMC8877938

[B142] Pandin C. Le Coq D. Deschamps J. Védie R. Rousseau T. Aymerich S.. (2018). Complete genome sequence of *Bacillus velezensis* QST713: A biocontrol agent that protects *Agaricus bisporus* crops against the green mould disease. J. Biotechnol. 278, 10–19. doi: 10.1016/j.jbiotec.2018.04.014 29702132

[B143] Park Y. G. Mun B. G. Kang S. M. Hussain A. Shahzad R. Seo C.-W.. (2017). *Bacillus aryabhattai* SRB02 tolerates oxidative and nitrosative stress and promotes the growth of soybean by modulating the production of phytohormones. PLos One 12, e0173203. doi: 10.1371/journal.pone.0173203 28282395 PMC5345817

[B144] Pereira S. I. A. Abreu D. Moreira H. Vega A. Castro P. M. L. (2020). Plant growth-promoting rhizobacteria (PGPR) improve the growth and nutrient use efficiency in maize (*Zea mays* l.) under water deficit conditions. Heliyon 6, e05106. doi: 10.1016/j.heliyon.2020.e05106 33083600 PMC7550905

[B145] Pieterse C. M. Zamioudis C. Berendsen R. L. Weller D. M. Van Wees S. C. Bakker P. A. (2014). Induced systemic resistance by beneficial microbes. Annu. Rev. Phytopathol. 52, 347–375. doi: 10.1146/annurev-phyto-082712-102340 24906124

[B146] Pinedo I. Ledger T. Greve M. Poupin M. J. (2015). *Burkholderia phytofirmans* PsJN induces long-term metabolic and transcriptional changes involved in *Arabidopsis thaliana* salt tolerance. Front. Plant Sci. 6. doi: 10.3389/fpls.2015.00466 PMC4477060 26157451

[B147] Rana S. Chauhan R. Walia A. Sharma G. D. Datt N. (2021). Beneficial microbes in agriculture under abiotic stress conditions: An overview. Pharma Innov. 10, 360–368. doi: 10.22271/tpi.2021.v10.i1e.5542

[B148] Rashid U. Yasmin H. Hassan M. N. Naz R. Nosheen A. Sajjad M.. (2022). Drought-tolerant *Bacillus megaterium* isolated from semi-arid conditions induces systemic tolerance of wheat under drought conditions. Plant Cell Rep. 41, 549–569. doi: 10.1007/s00299-020-02640-x 33410927

[B149] Ritonga F. N. Ngatia J. N. Wang Y. Khoso M. A. Farooq U. Chen S. (2021). AP2/ERF, an important cold stress-related transcription factor family in plants: A review. Physiol. Mol. Biol. Plants 27, 1953–1968. doi: 10.1007/s12298-021-01061-8 34616115 PMC8484489

[B150] Rouphael Y. Colla G. (2018). Synergistic biostimulatory action: Designing the next generation of plant biostimulants for sustainable agriculture. Front. Plant Sci. 9. doi: 10.3389/fpls.2018.01655 PMC6243119 30483300

[B151] Rouphael Y. Colla G. (2020). Toward a sustainable agriculture through plant biostimulants: From experimental data to practical applications. Agronomy 10, 1461. doi: 10.3390/agronomy10101461

[B152] Saechow S. Thammasittirong A. Kittakoop P. Prachya S. Thammasittirong S. N. R. (2018). Antagonistic activity against dirty panicle rice fungal pathogens and plant growth-promoting activity of *Bacillus amyloliquefaciens* BAS23. J. Microbiol. Biotechnol. 28, 1527–1535. doi: 10.4014/jmb.1804.04025 30369116

[B153] Saikia J. Sarma R. K. Dhandia R. Yadav A. Bharali R. Gupta V. K.. (2018). Alleviation of drought stress in pulse crops with ACC deaminase producing rhizobacteria isolated from acidic soil of northeast India. Sci. Rep. 8, 3560. doi: 10.1038/s41598-018-21921-w 29476114 PMC5824784

[B154] Saleem M. Fariduddin Q. Janda T. (2021a). Multifaceted role of salicylic acid in combating cold stress in plants: A review. J. Plant Growth Regul. 40, 464–485. doi: 10.1007/s00344-020-10152-x

[B155] Saleem S. Iqbal A. Ahmed F. Ahmad M. (2021b). Phytobeneficial and salt stress mitigating efficacy of IAA producing salt tolerant strains in. Gossypium hirsutum. Saudi J. Biol. Sci. 28, 5317–5324. doi: 10.1016/j.sjbs.2021.05.056 34466110 PMC8381066

[B156] Saltveit M. E. (2000). Wound induced changes in phenolic metabolism and tissue browning are altered by heat shock. Postharvest Biol. Technol. 21, 61–69. doi: 10.1016/S0925-5214(00)00165-4

[B157] Saravanakumar D. Kavino M. Raguchander T. Subbian P. Samiyappan R. (2011). Plant growth promoting bacteria enhance water stress resistance in green gram plants. Acta Physiol. Plant 33, 203–209. doi: 10.1007/s11738-010-0539-1

[B158] Sarkar J. Chakraborty U. Chakraborty B. (2021). High-temperature resilience in *Bacillus safensis* primed wheat plants: A study of dynamic response associated with modulation of antioxidant machinery, differential expression of HSPs and osmolyte biosynthesis. Environ. Exp. Bot. 182, 104315. doi: 10.1016/j.envexpbot.2020.104315

[B159] Sarkar A. Ghosh P. K. Pramanik K. Mitra S. Soren T. Pandey S.. (2018). A *halotolerant enterobacter* sp. displaying ACC deaminase activity promotes rice seedling growth under salt stress. Res. Microbiol. 169, 20–32. doi: 10.1016/j.resmic.2017.08.005 28893659

[B160] Sarma R. K. Saikia R. (2014). Alleviation of drought stress in mung bean by strain *Pseudomonas aeruginosa* GGRJ21. Plant Soil 377, 111–126. doi: 10.1007/s11104-013-1981-9

[B161] Sen S. Ghosh D. Mohapatra S. (2018). Modulation of polyamine biosynthesis in *Arabidopsis thaliana* by a drought mitigating *Pseudomonas putida* strain. Plant Physiol. Biochem. 129, 180–188. doi: 10.1016/j.plaphy.2018.05.034 29886249

[B162] Shabaan M. Asghar H. N. Zahir Z. A. Zhang X. Sardar M. F. Li H. (2022). Salt-tolerant PGPR confer salt tolerance to maize through enhanced soil biological health, enzymatic activities, nutrient uptake and antioxidant defense. Front. Microbiol. 13. doi: 10.3389/fmicb.2022.901865 PMC9136238 35633670

[B163] Shaffique S. Khan M. A. Wani S. H. Pande A. Imran M. Kang S. M.. (2022). A review on the role of endophytes and plant growth promoting rhizobacteria in mitigating heat stress in plants. Microorganisms 10, 1286. doi: 10.3390/microorganisms10071286 35889005 PMC9319882

[B164] Shahid M. Akram M. S. Khan M. A. Zubair M. Shah S. M. Ismail M.. (2018). A phytobeneficial strain *planomicrobium* sp. MSSA-10 triggered oxidative stress responsive mechanisms and regulated the growth of pea plants under induced saline environment. J. Appl. Microbiol. 124, 1566–1579. doi: 10.1111/jam.13732 29444380

[B165] Shahid M. Ameen F. Maheshwari H. S. Ahmed B. AlNadhari S. Khan M. S. (2021). Colonization of *Vigna radiata* by a halotolerant bacterium *Kosakonia sacchari* improves the ionic balance, stressor metabolites, antioxidant status and yield under NaCl stress. Appl. Soil Ecol. 158, 103809. doi: 10.1016/j.apsoil.2020.103809

[B166] Shahrajabian M. H. Chaski C. Polyzos N. Petropoulos S. A. (2021). Biostimulants application: A low input cropping management tool for sustainable farming of vegetables. Biomolecules 11, 698. doi: 10.3390/biom11050698 34067181 PMC8150747

[B167] Sivamani E. Bahieldin A. Wraith J. M. Al-Niemi T. Dyer W. E. Ho T. H. D.. (2000). Improved biomass productivity and water use efficiency under water deficit conditions in transgenic wheat constitutively expressing the barley HVA1 gene. Plant Sci. 155, 1–9. doi: 10.1016/s0168-9452(99)00247-2 10773334

[B168] Srivastava S. Bist V. Srivastava S. Singh P. C. Trivedi P. K. Asif M. H.. (2016). Unraveling aspects of *Bacillus amyloliquefaciens* mediated enhanced production of rice under biotic stress of *Rhizoctonia solani* . Front. Plant Sci. 7. doi: 10.3389/fpls.2016.00587 PMC4858605 27200058

[B169] Subramanian P. Kim K. Krishnamoorthy R. Mageswari A. Selvakumar G. Sa T. (2016). Cold stress tolerance in psychrotolerant soil bacteria and their conferred chilling resistance in tomato (*Solanum lycopersicum* mill.) under low temperatures. PLos One 11, e0161592. doi: 10.1371/journal.pone.0161592 27580055 PMC5006972

[B170] Su F. Jacquard C. Villaume S. Michel J. Rabenoelina F. Clément C.. (2015). *Burkholderia phytofirmans PsJN* reduces impact of freezing temperatures on photosynthesis in *Arabidopsis thaliana* . Front. Plant Sci. 6. doi: 10.3389/fpls.2015.00810 PMC4591482 26483823

[B171] Sunita K. Mishra I. Mishra J. Prakash J. Arora N. K. (2020). Secondary metabolites from halotolerant plant growth promoting rhizobacteria for ameliorating salinity stress in plants. Front. Microbiol. 11. doi: 10.3389/fmicb.2020.567768 PMC7641974 33193157

[B172] Swain P. Raman A. Singh S. P. Kumar A. (2017). Breeding drought tolerant rice for shallow rainfed ecosystem of eastern India. F. Crop Res. 209, 168–178. doi: 10.1016/j.fcr.2017.05.007 PMC5473176 28775653

[B173] Teklić T. Parađiković N. Špoljarević M. Zeljković S. Lončarić Z. Lisjak M. (2021). Linking abiotic stress, plant metabolites, biostimulants and functional food. Ann. Appl. Biol. 178, 169–191. doi: 10.1111/aab.12651

[B174] Tewari S. Arora N. K. (2018). Role of salicylic acid from *Pseudomonas aeruginosa* PF_23_ ^EPS+^ in growth promotion of sunflower in saline soils infested with phytopathogen *Macrophomina phaseolina* . Environ. Sustain. 1, 49–59. doi: 10.1007/s42398-018-0002-6

[B175] Theocharis A. Bordiec S. Fernandez O. Paquis S. Dhondt-Cordelier S. Baillieul F.. (2012). *Burkholderia phytofirmans* PsJN primes *Vitis vinifera* l. and confers a better tolerance to low nonfreezing temperatures. Mol. Plant Microbe Interact. 25, 241–249. doi: 10.1094/MPMI-05-11-0124 21942451

[B176] Tirry N. Kouchou A. Laghmari G. Lemjereb M. Hnadi H. Amrani K.. (2021). Improved salinity tolerance of *Medicago sativa* and soil enzyme activities by PGPR. Biocatal. Agric. Biotechnol. 31, 101914. doi: 10.1016/j.bcab.2021.101914

[B177] Tiryaki D. Aydın İ. ATICI Ö. (2018). Psychrotolerant bacteria isolated from the leaf apoplast of cold-adapted wild plants improve the cold resistance of bean (*Phaseolus vulgaris* l.) under low temperature. Cryobiology 86, 111–119. doi: 10.1016/j.cryobiol.2018.11.001 30419217

[B178] Torres R. O. Henry A. (2018). Yield stability of selected rice breeding lines and donors across conditions of mild to moderately severe drought stress. F. Crop Res. 220, 37–45. doi: 10.1016/j.fcr.2016.09.011 PMC5891920 29725159

[B179] Turan M. Güllüce M. Çakmak R. Şahin F. (2013). Effect of plant growth-promoting rhizobacteria strain on freezing injury and antioxidant enzyme activity of wheat and barley. J. Plant Nutr. 36, 731–748. doi: 10.1080/01904167.2012.754038

[B180] Vimal S. R. Patel V. K. Singh J. S. (2019). Plant growth promoting *Curtobacterium albidum* strain SRV4: An agriculturally important microbe to alleviate salinity stress in paddy plants. Ecol. Indic. 105, 553–562. doi: 10.1016/j.ecolind.2018.05.014

[B181] Vlot A. C. Sales J. H. Lenk M. Bauer K. Brambilla A. Sommer A.. (2021). Systemic propagation of immunity in plants. New Phytol. 229, 1234–1250. doi: 10.1111/nph.16953 32978988

[B182] Vocciante M. Grifoni M. Fusini D. Petruzzelli G. Franchi E. (2022). The role of plant growth-promoting rhizobacteria (PGPR) in mitigating plant’s environmental stresses. Appl. Sci. 12, 1231. doi: 10.3390/app12031231

[B183] Wang G. Xu X. Wang H. Liu Q. Yang X. Liao L.. (2019). A tomato transcription factor, SlDREB3 enhances the tolerance to chilling in transgenic tomato. *Plant physiol* . Biochem 142, 254–262. doi: 10.1016/j.plaphy.2019.07.017 31326718

[B184] Wan L. J. Tian Y. He M. Zheng Y. Q. Lyu Q. Xie R. J.. (2021). Effects of chemical fertilizer combined with organic fertilizer application on soil properties, citrus growth physiology, and yield. Agriculture 11, 1207. doi: 10.3390/agriculture11121207

[B185] Wei Y. Chen H. Wang L. Zhao Q. Wang D. Zhang T. (2022). Cold acclimation alleviates cold stress-induced PSII inhibition and oxidative damage in tobacco leaves. Plant Signal. Behav. 17, 2013638. doi: 10.1080/15592324.2021.2013638 34964430 PMC8920150

[B186] Win K. T. Tanaka F. Okazaki K. Ohwaki Y. (2018). The ACC deaminase expressing endophyte *Pseudomonas* spp. enhances NaCl stress tolerance by reducing stress-related ethylene production, resulting in improved growth, photosynthetic performance, and ionic balance in tomato plants. Plant Physiol. Biochem. 127, 599–607. doi: 10.1016/j.plaphy.2018.04.038 29730579

[B187] Woo O. G. Kim H. Kim J. S. Keum H. L. Lee K. C. Sul W. J.. (2020). *Bacillus subtilis* strain GOT9 confers enhanced tolerance to drought and salt stresses in *Arabidopsis thaliana* and brassica campestris. Plant Physiol. Biochem. 148, 359–367. doi: 10.1016/j.plaphy.2020.01.032 32018064

[B188] Wu Y. Zhou J. Li C. Ma Y. (2019). Antifungal and plant growth promotion activity of volatile organic compounds produced by *Bacillus amyloliquefaciens* . MicrobiologyOpen 8, e00813. doi: 10.1002/mbo3.813 30907064 PMC6692555

[B189] Yasmin H. Bano A. Wilson N. L. Nosheen A. Naz R. Hassan M. N.. (2022). Drought-tolerant pseudomonas sp. showed differential expression of stress-responsive genes and induced drought tolerance in *Arabidopsis thaliana* . Physiol. Plant 174, e13497. doi: 10.1111/ppl.13497 34245030

[B190] Yasmin H. Mazher J. Azmat A. Nosheen A. Naz R. Hassan M. N.. (2021b). Combined application of zinc oxide nanoparticles and biofertilizer to induce salt resistance in safflower by regulating ion homeostasis and antioxidant defence responses. Ecotoxicol Environ. Saf. 218, 112262. doi: 10.1016/j.ecoenv.2021.112262 33964549

[B191] Yasmin H. Naeem S. Bakhtawar M. Jabeen Z. Nosheen A. Naz R.. (2020). Halotolerant rhizobacteria *Pseudomonas pseudoalcaligenes* and *Bacillus subtilis* mediate systemic tolerance in hydroponically grown soybean (*Glycine max* l.) against salinity stress. PLos One 15, e0231348. doi: 10.1371/journal.pone.0231348 32298338 PMC7162512

[B192] Yasmin H. Rashid U. Hassan M. N. Nosheen A. Naz R. Ilyas N.. (2021a). Volatile organic compounds produced by *Pseudomonas pseudoalcaligenes* alleviated drought stress by modulating defense system in maize (*Zea mays* l.). Physiol. Plant 172, 896–911. doi: 10.1111/ppl.13304 33314151

[B193] Yu Y. Gui Y. Li Z. Jiang C. Guo J. Niu D. (2022). Induced systemic resistance for improving plant immunity by beneficial microbes. Plants 11, 386. doi: 10.3390/plants11030386 35161366 PMC8839143

[B194] Yu T. F. Xu Z. S. Guo J. K. Wang Y. X. Abernathy B. Fu J. D.. (2017). Improved drought tolerance in wheat plants overexpressing a synthetic bacterial cold shock protein gene *SeCspA* . Sci. Rep. 7, 44050. doi: 10.1038/srep44050 28281578 PMC5345034

[B195] Zandalinas S. I. Mittler R. Balfagón D. Arbona V. Gómez-Cadenas A. (2018). Plant adaptations to the combination of drought and high temperatures. Physiol. Plant 162, 2–12. doi: 10.1111/ppl.12540 28042678

[B196] Zang X. Xiaoli G. Wang F. Liu Z. Zhang L. Zhao Y.. (2017). Overexpression of wheat ferritin gene *TaFER-5B* enhances tolerance to heat stress and other abiotic stresses associated with the ROS scavenging. BMC Plant Biol. 17, 1–13. doi: 10.1186/s12870-016-0958-2 28088182 PMC5237568

[B197] Zarei T. Moradi A. Kazemeini S. A. Akhgar A. Rahi A. A. (2020). The role of ACC deaminase producing bacteria in improving sweet corn (*Zea mays* l. var saccharata) productivity under limited availability of irrigation water. Sci. Rep. 10, 1–12. doi: 10.1038/s41598-020-77305-6 33230222 PMC7683742

[B198] Zebelo S. Song Y. Kloepper J. W. Fadamiro H. (2016). Rhizobacteria activates (+)-δ-cadinene synthase genes and induces systemic resistance in cotton against beet armyworm (*Spodoptera exigua*). Plant Cell Environ. 39, 935–943. doi: 10.1111/pce.12704 26715260

[B199] Zhou Y. Sommer M. L. Hochholdinger F. (2021). Cold response and tolerance in cereal roots. J. Exp. Bot. 72, 7474–7481. doi: 10.1093/jxb/erab334 34270744

[B200] Zubair M. Hanif A. Farzand A. Sheikh T. M. M. Khan A. R. Suleman M.. (2019). Genetic screening and expression analysis of *psychrophilic bacillus* spp. reveal their potential to alleviate cold stress and modulate phytohormones in wheat. Microorganisms 7, 337. doi: 10.3390/microorganisms7090337 31510075 PMC6780275

